# Penalized variable selection in multi-parameter regression survival modeling

**DOI:** 10.1177/09622802231203322

**Published:** 2023-10-12

**Authors:** Fatima-Zahra Jaouimaa, Il Do Ha, Kevin Burke

**Affiliations:** 1 Department of Mathematics and Statistics, 8808 University of Limerick, Ireland; 2 Department of Statistics, 34998 Pukyong National University, Busan, South Korea

**Keywords:** Variable selection, multi-parameter regression, Weibull, penalized maximum likelihood, differential evolution algorithm

## Abstract

Standard survival models such as the proportional hazards model contain a single regression component, corresponding to the scale of the hazard. In contrast, we consider the so-called “multi-parameter regression” approach whereby covariates enter the model through multiple distributional parameters simultaneously, for example, scale and shape parameters. This approach has previously been shown to achieve flexibility with relatively low model complexity. However, beyond a stepwise type selection method, variable selection methods are underdeveloped in the multi-parameter regression survival modeling setting. Therefore, we propose penalized multi-parameter regression estimation procedures using the following penalties: least absolute shrinkage and selection operator, smoothly clipped absolute deviation, and adaptive least absolute shrinkage and selection operator. We compare these procedures using extensive simulation studies and an application to data from an observational lung cancer study; the Weibull multi-parameter regression model is used throughout as a running example.

## 1. Introduction

The most popular regression model for censored survival data is Cox’s proportional hazards (PH) model,^1^ with a hazard function given by $h(t|\tau_{i},\gamma )=\tau_{i}h_{0}(t|\gamma )$ , where $\tau_{i}$ is a scale parameter that varies per individual i=1,…,n , and γ is a shape parameter common to all individuals in which case $h_{0}$ is referred to as a baseline hazard function. Its appeal is due to the fact that covariate effects can be estimated in the form of relative risks, without specializing to any particular underling hazard shape, that is, $h_{0}$ is an unspecified function whose shape is a nuisance parameter. Such is its popularity, the model is often used without critical assessment of the fundamental PH assumption.^2^ One of the most common approaches for dealing with a covariate that does not follow this assumption is through stratification of the hazard function, whereby a different baseline hazard is assumed for different levels of the covariate in question. However, issues with this approach are that: it is limited to categorical variables so that application to continuous variables requires ad-hoc binning, stratification with respect to multiple variables and/or variables with many levels leads to efficiency losses due to small number of individuals in the sub-groups, and the effects of stratified variables (via relative risks) are not estimated as they are absorbed into the “nuisance” hazard shape (see Therneau and Grambsch^3^ and references therein).

Rather than treating the hazard shape as a nuisance, we make use of a fully parametric hazard function characterized by multiple distributional parameters. In the two-parameter case that we focus on in this article, the hazard has the form $h(t|\tau_{i},\gamma_{i})=\tau_{i}h_{0}(t;\gamma_{i})$ , where $\tau_{i}$ is the scale parameter for the hazard (as in the Cox model) and $\gamma_{i}$ is its shape, which now also varies per individual (hence, $h_{0}$ is no longer a baseline hazard function). Importantly, both the scale *and* the shape parameters depend on individual covariates (via the subscript i ), an activity that we refer to as multi-parameter regression (MPR) modeling^4^; the approach may also be referred to as distributional regression^5^ (see also Rigby and Stasinopoulos^6^ and Stasinopoulos et al.^7^). The *multi*-parameter regression nomenclature distinguishes our approach from classical models where there is just *one* regression component such as the hazard scale parameter in a Cox model, or the location parameter in a generalized linear model.^8^

**Figure 1.**
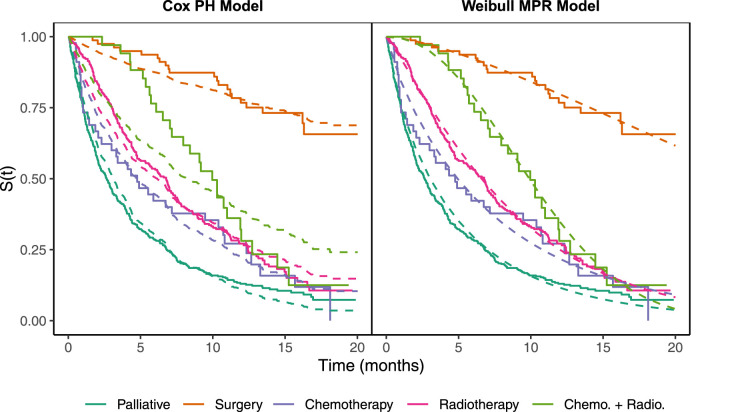
Kaplan–Meier curves (solid) for different treatment groups with model-based curves overlaid (dashed) for the Cox PH model (left) and Weibull MPR model (right). PH: proportional hazards; MPR: multi-parameter regression.

As a motivating example, Figure 1 displays data from a lung cancer study used in Burke and MacKenzie,^4^ where it is clear that the MPR model has the flexibility to adapt to the different distributional shapes evident across the treatment groups (see Section 5 for a multi-factor analysis of this dataset). Indeed, Burke and MacKenzie^4^ explored the general use of MPR models in the survival context, demonstrating the usefulness of jointly modeling the scale and shape of the Weibull distribution. Earlier examples of MPR models in survival analysis include a location-dispersion extension of the Weibull accelerated failure time (AFT) model,^9^ and first-hitting-time models with covariate-dependent drift and initial-state parameters.^10,11^ More recently, MPR survival models have been developed further through the incorporation of frailty effects in interval-censored data,^12^ the use of the adapted power generalized Weibull model,^13^ which is more general than the Weibull and has also been extended to handle bivariate data,^14^ and a semi-parametric extension of the AFT model.^15^

Regardless of the modeling approach taken (MPR or otherwise), a commonly encountered challenge in statistical applications is the selection of a subset of explanatory variables of interest,^16,17^ that is, the elimination of unimportant variables to yield simpler, more explainable models. However, the literature in this area is somewhat lacking for MPR survival models beyond a stepwise procedure developed by Burke and MacKenzie.^4^ Due to the inherent discreteness of stepwise procedures (i.e., covariates are either “in” or “out”), they may be unstable in terms of the selected model.^18^ Furthermore, they can be computationally demanding since, with p covariates, there are $2^{p}$ submodels—and this issue is more acute in the MPR setting where there are multiple regression components, for example, in the scale-shape model, there are $2^{2p}$ submodels. On the other hand, more modern approaches based on penalization carry out estimation and (continuous) model selection simultaneously, and are capable of handling a larger number of covariates in a more stable and efficient manner, for example, the least absolute shrinkage and selection operator (LASSO),^19^ the smoothly clipped absolute deviation (SCAD),^20^ and the adaptive LASSO (ALASSO).^21^

To the best of our knowledge, the use of the aforementioned penalized procedures for MPR survival models is lacking. Given that classical statistical models contain only one regression component, it is not unexpected that the penalized estimation literature is focused around this setting.^22^ An exception is the work of Groll et al.^23^ who develop LASSO-type penalization for generalized additive models for location, scale and shape (GAMLSS) albeit not in a survival context. Therefore, the aim of this article is to develop such procedures for MPR survival models. More specifically, we use the Weibull MPR model as an example, develop gradient-based estimation procedures for LASSO, SCAD, and ALASSO by using a smooth approximation to the absolute value function,^24–26^ and investigate the need for a separate tuning parameter for each regression component. Tuning parameter selection is carried out using a Bayesian information criterion (BIC) function, where, due to the intensivity of grid search when there are multiple regression components (as noted in the GAMLSS context^23^), we make use of a differential evolution “global” optimization procedure^27,28^ to explore the tuning parameter space.

The remainder of this article is organized as follows. In Section 2, we present the Weibull MPR model, the penalty functions, and the penalized likelihood estimation procedure. Section 3 describes the model estimation and inference procedure along with the algorithm for selecting tuning parameters. Simulation studies are provided in Section 4 where we evaluate the performance of the proposed methods, and these methods are then applied to data from an observational study of patients with lung cancer in Section 5. We conclude with some final remarks in Section 6.

## 2. Model formulation

### 2.1. Weibull MPR model

Although the variable selection methods we consider in this article can be applied to any parametric MPR model, it is helpful to focus on a specific example. We, therefore, consider the Weibull MPR model since the Weibull distribution is one of the most popular parametric survival distributions. In this case, the hazard function for survival time ${\tilde{T}}_{i}$ corresponding to the i th individual is given by

$$h(t|\tau_{i},\gamma_{i})=\tau_{i}\gamma_{i}t^{\gamma_{i}-1}$$

for i=1,2,…,n , where $\tau_{i}>0$ and $\gamma_{i}>0$ are the *covariate-dependent* scale and shape parameters, respectively. This is an MPR model by virtue of both distributional parameters depending on covariates, and we specify these regression components as follows:

$$\log\;(\tau_{i})=x_{i}^{T}\beta ,\;\log\;(\gamma_{i})=z_{i}^{T}\alpha$$

where $x_{i}=(1,x_{i1},\ldots ,x_{ip}{)}^{T}$ and $z_{i}=(1,z_{i1},\ldots ,z_{iq}{)}^{T}$ are scale and shape covariate vectors which may or may not have covariates in common, $\beta =(\beta_{0},\beta_{1},\ldots ,\beta_{p}{)}^{T}$ and $\alpha =(\alpha_{0},\alpha_{1},\ldots ,\alpha_{q}{)}^{T}$ are the corresponding regression coefficients, and the log link is used to ensure positivity of the parameters.

Without the loss of generality, we consider the ratio of hazards under this model for individuals i and $i^{\prime }$ who we assume have identical covariate profiles apart from the first covariate in the scale and shape vectors, where $x_{i1}=z_{i1}=c+1$ and $x_{i^{\prime }1}=z_{i^{\prime }1}=c$ , that is, the first covariate differs by one unit for these individuals. Given this setup, $\tau_{i}=\exp\;(\beta_{1})\tau_{i^{\prime }}$ and $\gamma_{i}=\exp\;(\alpha_{1})\gamma_{i^{\prime }}$ , and the hazard ratio is

$$\frac{h(t|\tau_{i},\gamma_{i})}{h(t|\tau_{i^{\prime }},\gamma_{i^{\prime }})}=\exp\;(\beta_{1}+\alpha_{1})t^{\exp\;(z_{i^{\prime }}^{T}\alpha )\{\exp\;(\alpha_{1})-1\}},$$

where $z_{i^{\prime }}^{T}=(1,c,z_{i2},\ldots ,z_{iq})$ , that is, the vector $z_{i}$ with the element $z_{i1}$ fixed at the value c . The dependence on $z_{i^{\prime }}$ is typically dealt with through the use of a representative covariate profile such as the empirical modal or mean values.^4^ Importantly, when $\alpha_{1}=0$ , the hazard ratio reduces to $\exp\;(\beta_{1})$ , which is the typical constant hazard ratio from a PH (Cox) model.

Parameter estimation within the unpenalized MPR model can be carried out in a standard fashion using maximum likelihood. First, let $T_{i}=\text{min}({\tilde{T}}_{i},C_{i})$ be the observed survival time for the i th individual. Then the associated log-likelihood function is given by

(1) $$\ell_{0}(\theta )=\sum_{i=1}^{n}\delta_{i}\{\log\;\tau_{i}+\log\;\gamma_{i}+(\gamma_{i}-1)\log\;t_{i}\}-\tau_{i}t_{i}^{\gamma_{i}}$$

where $\theta =(\beta^{T},\alpha^{T}{)}^{T}$ is the full parameter vector, $t_{i}$ is the realization of $T_{i}$ , and $\delta_{i}$ is the censoring indicator which takes the value 0 for censored survival times and 1 for uncensored survival times. Beyond the Weibull case we consider here, the likelihood function is $\sum_{i=1}^{n}\delta_{i}\log\;h(t_{i}|x_{i},z_{i})-H(t_{i}|x_{i},z_{i})$ , where $H(t|x_{i},z_{i})=\int_{0}^{t}h(u|x_{i},z_{i})du$ is the cumulative hazard function.

### 2.2. Penalized likelihood

Penalized MPR estimation can be developed on the basis of maximizing a penalized log-likelihood given by

(2) $$\ell (\theta )=\ell_{0}(\theta )-n\sum_{j=0}^{p}J_{\lambda_{\beta_{j}}}(|\beta_{j}|)-n\sum_{j=0}^{q}J_{\lambda_{\alpha_{j}}}(|\alpha_{j}|)$$

where $\ell_{0}(\theta )$ is the unpenalized likelihood, $\lambda =(\lambda_{\beta_{0}},\lambda_{\beta_{1}},\ldots ,\lambda_{\beta_{p}},\lambda_{\alpha_{0}},\lambda_{\alpha_{1}},\ldots ,\lambda_{\alpha_{q}})$ is a vector of coefficient-specific tuning parameters, and $J_{\lambda_{\beta_{j}}}(\cdot )$ and $J_{\lambda_{\alpha_{j}}}(\cdot )$ are scale and shape penalty functions which we assume have the same functional form (but differ with respect to the tuning parameter). As is standard practice, we assume that the intercepts are not penalized, and, therefore, define $\lambda_{\beta_{0}}\equiv \lambda_{\alpha_{0}}\equiv 0$ . We also assume that covariates are standardized so that penalization is independent of the particular units of measurement; in all of our numerical work, we carry out this standardization internally and present regression coefficients on the original scale.

Although we have defined λ quite generally, we will in fact impose constraints on this vector (beyond fixing $\lambda_{\beta_{0}}\equiv \lambda_{\alpha_{0}}\equiv 0$ ) by considering the following possibilities (for j≠0 ):

(i) single penalty,
$$\lambda_{\beta_{j}}=\lambda_{\alpha_{j}}=\lambda$$
(ii) single adaptive penalty,
$$\lambda_{\beta_{j}}=\lambda w_{\beta_{j}},\;\lambda_{\alpha_{j}}=\lambda w_{\alpha_{j}}$$
  where $w_{\beta_{j}}$ and $w_{\alpha_{j}}$ are predefined weights,
(iii) separate non-adaptive penalties,
$$\lambda_{\beta_{j}}=\lambda_{\beta },\;\lambda_{\alpha_{j}}=\lambda_{\alpha }$$
(iv) separate adaptive penalties,
$$\lambda_{\beta_{j}}=\lambda_{\beta }w_{\beta_{j}},\;\lambda_{\alpha_{j}}=\lambda_{\alpha }w_{\alpha_{j}}$$

(i) and (ii) are standard approaches where a single penalty, λ , applies to the whole vector of parameters. This is reasonable in a standard setting where there is only a β vector. However, in this particular MPR setting, we have two separate distributional parameters, which exist on different scales. For this reason, we investigate methods (iii) and (iv) which apply different penalties to the two regression vectors via $\lambda_{\beta }$ and $\lambda_{\alpha }$ .

For the purpose of this article, we consider the most commonly used penalties, namely the LASSO,^19^

$$J_{\lambda_{\theta_{j}}}(|\theta_{j}|)=\lambda_{\theta_{j}}|\theta_{j}|$$

which although popular, is known to select too many variables^29^; the non-convex SCAD,^20^

$$J_{\lambda_{\theta_{j}}}(|\theta_{j}|)=\left\{ \begin{array}{ll} \lambda_{\theta_{j}}(|\theta_{j}|) & \;\text{if }\;|\theta_{j}|\leq \lambda_{\theta_{j}}\\ \frac{2a\lambda_{\theta_{j}}|\theta_{j}|-\theta_{j}^{2}-\lambda_{\theta_{j}}^{2}}{2(a-1)} & \;\text{if }\;\lambda_{\theta_{j}}<|\theta_{j}|<a\lambda_{\theta_{j}}\\ \frac{\lambda_{\theta_{j}}^{2}(a+1)}{2} & \;\text{if }\;|\theta_{j}|\geq a\lambda_{\theta_{j}} \end{array}\right.$$

where a=3.7 , and the ALASSO,^21^

$$J_{\lambda_{\theta_{j}}}(|\theta_{j}|)=\lambda_{\theta_{j}}w_{\theta_{j}}|\theta_{j}|$$

where typically, $w_{\theta_{j}}=1/|{\hat{\theta }}_{0,j}|$ and ${\hat{\theta }}_{0,j}$ is an unpenalized estimate of $\theta_{j}$ . These so-called adaptive weights are used to apply different penalties to different regression coefficients such that a larger amount of shrinkage is applied to the unimportant variables. Here, we use $\theta_{j}$ to denote a generic regression coefficient, and $\lambda_{\theta_{j}}$ is the corresponding tuning parameter. Note that the LASSO and SCAD are non-adaptive, and, therefore, relate to options (i) and (iii) above, whereas the ALASSO relates to options (ii) and (iv). SCAD and the ALASSO are known to possess the oracle property, that is, the procedure asymptotically identifies the right subset model and estimates the coefficients and covariance matrix as though the true model was known in advance.^20^ Fan and Li^20^ found the choice of *a* = 3.7 to give very good practical performance for various variable selection problems, and as a result this value has been widely used throughout the literature.^30–33^

## 3. Penalized estimation procedure

### 3.1. Model fitting

We define

(3) $$\hat{\theta }=\underset{\theta }{\text{argmax}}\;\ell (\theta )$$

where ℓ(θ) is given by (2). The corresponding score functions are given by

(4) $$\begin{array}{rl} \frac{\partial \ell }{\partial \beta } & =\frac{\partial \ell_{0}}{\partial \beta }-nV_{\beta }=X^{T}U_{\beta }-nV_{\beta }\\ \frac{\partial \ell }{\partial \alpha } & =\frac{\partial \ell_{0}}{\partial \alpha }-nV_{\alpha }=Z^{T}U_{\alpha }-nV_{\alpha } \end{array}$$

where X is an n×(p+1) matrix whose i th row is $x_{i}$ , Z is an n×(q+1) matrix whose i th row is $z_{i}$ ; $U_{\beta }$ and $U_{\alpha }$ are vectors of length n such that $U_{\beta i}=\delta_{i}-\tau_{i}t_{i}^{\gamma_{i}}$ and $U_{\alpha i}=\delta_{i}(1+\gamma_{i}\log\;t_{i})-\tau_{i}\gamma_{i}t_{i}^{\gamma_{i}}\log\;t_{i}$ ; $V_{\beta }$ and $V_{\alpha }$ are vectors of lengths p+1 and q+1 , respectively, such that, for j≥0 , $V_{\beta ,j+1}=dJ_{\lambda_{\beta_{j}}}(|\beta_{j}|)/d\beta_{j}=J_{\lambda_{\beta_{j}}}^{\prime }(|\beta_{j}|)d|\beta_{j}|/d\beta_{j}$ and $V_{\alpha ,j+1}=dJ_{\lambda_{\alpha_{j}}}(|\alpha_{j}|)/d\alpha_{j}=J_{\lambda_{\alpha_{j}}}^{\prime }(\left|\alpha_{j}\right|)d|\alpha_{j}|/d\alpha_{j}$ .

Note however, the presence of the absolute value function renders the penalty functions non-differentiable at zero. Various algorithms have been developed to overcome this issue including quadratic programing,^19^ least-angle regression (LARS),^34^ co-ordinate descent,^35^ and the local quadratic approximation.^20^ In this article, we take a similar approach to that of Hunter and Li,^24^ Oelker and Tutz,^25^ and Lloyd-Jones et al.,^26^ and use an extension of the absolute value function given by

$$a(x)=\sqrt{x^{2}+\epsilon^{2}}-\epsilon$$

where $\lim_{\epsilon \to 0}a(x)=|x|$ . This yields a differentiable penalty so that standard gradient-based optimization algorithms can be applied straightforwardly and transparently. Thus, $a^{\prime }(x)=x/\sqrt{\epsilon^{2}+x^{2}}$ (which is an approximation of the signum function) and $a^{\prime \prime }(x)=\epsilon^{2}/(\epsilon^{2}+x^{2}{)}^{3/2}$ . Smaller values of ϵ bring the approximate penalty closer to the original penalty, but also closer to the penalty being non-differentiable; we have found that fixing $\epsilon =10^{-4}$ generally works well. As we use smooth J(⋅) functions, and a(x) in place of |x| , (4) is then smooth in the parameters and can therefore be solved using the Netwon-Raphson algorithm.

We denote by I(θ) the matrix of second derivatives of ℓ(θ) , that is, $-\nabla_{\theta }\nabla_{\theta }^{T}\ell (\theta )$ . Then,

$$\begin{array}{r} I(\theta )=I_{0}(\theta )+\left(\begin{array}{cc} n\Sigma_{\beta } & 0\\ 0 & n\Sigma_{\alpha } \end{array}\right)=\left(\begin{array}{cc} X^{T}W_{\beta }X+n\Sigma_{\beta } & X^{T}W_{\alpha \beta }Z\\ Z^{T}W_{\alpha \beta }X & Z^{T}W_{\alpha }Z+n\Sigma_{\alpha } \end{array}\right) \end{array}$$

where $I_{0}(\theta )=-\nabla_{\theta }\nabla_{\theta }^{T}\ell_{0}(\theta )$ is the usual observed information matrix of the unpenalized likelihood; $\Sigma_{\beta }$ and $\Sigma_{\alpha }$ appear due to the penalties, and are diagonal matrices of dimension (p+1)×(p+1) and (q+1)×(q+1) , respectively, such that, for j≥0 , $\Sigma_{\beta ,j+1,j+1}=d^{2}J_{\lambda_{\beta_{j}}}(|\beta_{j}|)/{d\beta_{j}}^{2}$ and $\Sigma_{\alpha ,j+1,j+1}=d^{2}J_{\lambda_{\alpha_{j}}}(|\alpha_{j}|)/{d\alpha_{j}}^{2}$ ; and $W_{\beta }$ , $W_{\alpha }$ , and $W_{\alpha \beta }$ are n×n diagonal matrices whose i th diagonal elements are given by $\tau_{i}t_{i}^{\gamma_{i}}$ , $\{\tau_{i}t_{i}^{\gamma_{i}}(\gamma_{i}\log\;t_{i}+1)-\delta_{i}\}\gamma_{i}\log\;t_{i}$ , and $\tau_{i}\gamma_{i}t_{i}^{\gamma_{i}}\log\;t_{i}$ , respectively. Thus, following Ha et al.,^36^ the resulting system of Newton-Raphson equations, which are iteratively solved for $\theta^{\left(m+1\right)}=({\beta^{\left(m+1\right)}}^{T},{\alpha^{\left(m+1\right)}}^{T}{)}^{T}$ , can be written compactly as

(5) $$\left(\begin{array}{cc} X^{T}W_{\beta }^{\left(m\right)}X+n\Sigma_{\beta }^{\left(m\right)} & X^{T}W_{\alpha \beta }^{\left(m\right)}Z\\ Z^{T}W_{\alpha \beta }^{\left(m\right)}X & Z^{T}W_{\alpha }^{\left(m\right)}Z+n\Sigma_{\alpha }^{\left(m\right)} \end{array}\right)\left(\begin{array}{c} \beta^{\left(m+1\right)}-\beta^{\left(m\right)}\\ \alpha^{\left(m+1\right)}-\alpha^{\left(m\right)} \end{array}\right)=\left(\begin{array}{c} X^{T}U_{\beta }^{\left(m\right)}-nV_{\beta }^{\left(m\right)}\\ Z^{T}U_{\alpha }^{\left(m\right)}-nV_{\alpha }^{\left(m\right)} \end{array}\right)$$

where the various elements superscripted by (m) depend on $\theta^{\left(m\right)}$ , but this dependence is suppressed for notational convenience; we use the unpenalized estimates as the initial values in this iterative procedure, that is, $\theta^{\left(0\right)}={\hat{\theta }}_{0}$ . Having obtained the penalized estimates, $\hat{\theta }$ , the covariance can be estimated using the sandwich formula.^20,37,36,38^

(6) $$\hat{cov}(\hat{\theta })=\{I(\hat{\theta }){\}}^{-1}I_{0}(\hat{\theta })\{I(\hat{\theta }){\}}^{-1}$$

This formula is known to have good accuracy when the sample size is moderate,^20,37^ and its performance in our MPR setting is investigated in Section 4 through simulation studies.

**Figure 2.**
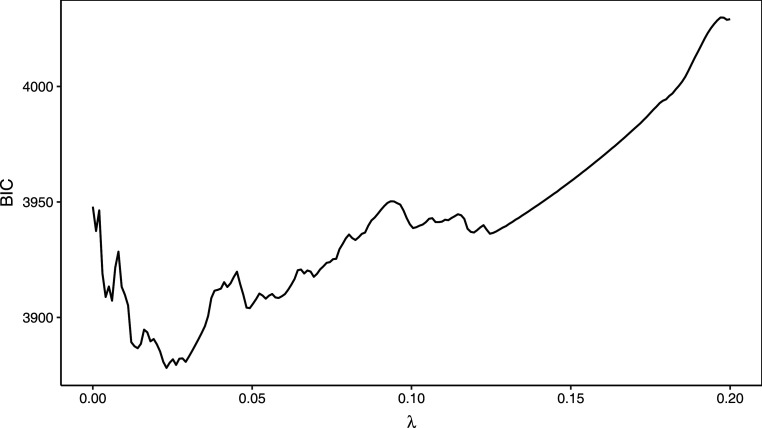
The BIC function evaluated at different tuning parameter values for the Weibull MPR model with the one tuning parameter LASSO penalty for the lung cancer data analysed in Section 5. The equivalent plot for the two tuning parameter LASSO penalties can be found in the Supplemental Material. BIC: Bayesian information criterion; MPR: multi-parameter regression;LASSO: least absolute shrinkage and selection operator.

### 3.2. Tuning parameter selection

The selection of the optimal tuning parameter(s) is typically done through the use of data-driven criteria such as generalized cross-validation (GCV), Akaike information criterion (AIC), or BIC. GCV and the AIC are known to be less efficient and selection inconsistent as model selection criteria.^39–41^ Wang et al.^42^ provided a formal proof that the shrinkage or tuning parameter selected using GCV may not be able to identify the true model consistently for the SCAD estimator in linear models and partially linear models. Instead, they suggest using the BIC and prove its model selection consistency property. A similar conclusion has been reached by Wang and Leng^43^ for the ALASSO. Hence, due to its widely reported superior empirical performance in variable selection, we use a BIC-type criterion to determine the values of the tuning parameter(s), where

(7) $$\text{BIC}(\lambda )=-2\ell_{0}(\hat{\theta })+df\cdot \log\;n$$

$\ell_{0}(\hat{\theta })$ is the unpenalized likelihood function defined in (1), n is the sample size, and $df=\text{tr}[\{I(\hat{\theta }){\}}^{-1}I_{0}(\hat{\theta })]$ is the effective degrees of freedom.^44^ The BIC is a function of λ through $\hat{\theta }$ and df , but for notational convenience, we suppress this dependence. We define

(8) $$\lambda^{*}=\underset{\lambda }{\text{argmin}}\;\text{BIC}(\lambda )$$

Note that, as described in Section 2.2, $\lambda^{*}$ will either be one-dimensional (when a common penalty is applied to β and α ) or two-dimensional (when separate penalties are applied). We define ${\hat{\theta }}^{*}$ to be the vector of coefficients values corresponding to $\lambda^{*}$ from (8).

The simplest method to solve this optimization problem is grid search. While it is straightforward to implement, grid search is known to suffer from the curse of dimensionality, that is, the number of grid points grows exponentially with the dimension. Furthermore, if the grid is too coarse, the minimum may be overlooked. This is especially true in the case of a multi-modal function, such as the BIC objective function as shown in Figure 2. This multi-modality arises in the BIC due to the tradeoff between complexity ( df ) and data fit ( $\ell_{0}(\hat{\theta })$ ). As an example, Table 1 “zooms in” on a particular portion of the BIC function from Figure 2, wherein the estimated $\beta_{6}$ coefficient decreases by an order of magnitude (becoming close to zero). Typically, df decreases rapidly when a given coefficient gets close to zero, but in such a way that the decreasing likelihood leads to a local minima in the BIC (at λ=0.023 in Table 1).

**Table 1.** The degrees of freedom, likelihood function, and BIC value evaluated at different tuning parameter values for the model with the LASSO penalty (one tuning parameter case) for the lung cancer dataset analysed in Section 5.

λ	*df*	ℓ	BIC	$\beta_{0}$	…	$\beta_{6}$	…
0.0221	34.00	−1825.3	3880.1	−2.7096		−0.0152	
0.0226	33.50	−1825.9	3877.9	−2.7034		−0.0133	
0.0230	33.30	−1826.6	3877.8	−2.6973		−0.0114	
0.0234	33.27	−1827.1	3878.8	−2.6915		−0.0095	
0.0238	33.23	−1827.7	3879.7	−2.6857		−0.0075	
0.0243	33.20	−1828.3	3880.7	−2.6799		−0.0056	
0.0247	33.15	−1828.9	3881.7	−2.6742		−0.0037	
0.0251	33.00	−1829.6	3882.0	−2.6684		−0.0019	

BIC: Bayesian information criterion; *df*: degrees of freedom; LASSO: least absolute shrinkage and selection operator.

Although the BIC’s consistency property has led to its extensive use in tuning-parameter selection, we suggest that such a multi-modal function would be better optimized by a “global” optimizer (rather than grid search as is typically used in the literature). In an empirical comparison of a wide variety of (stochastic and deterministic) algorithms for continuous global optimization, Mullen^28^ found DEoptim (implemented in R)^27^ to be among the best. The function implements a differential evolution algorithm, an example of an evolutionary strategy developed by Storn and Price^45^ (see Mullen et al.^27^ for a detailed overview of the underlying algorithm). As highlighted by an anonymous reviewer, model-based (Bayesian) optimization (MBO) lends itself well to situations where the objective function is computationally burdensome, for example, embeded within each BIC computation in (7) is a model estimated by iteratively solving the equations in (5) for a given λ vector. Although not considered by Mullen,^28^ our initial testing suggests that DEoptim also outperforms MBO as implemented in the mlrMBO package^46^ (see Supplemental Material).

### 3.3. Variable selection algorithm

The variable selection algorithm described above is summarized in the following points:

• *Initialization.* Set $\theta^{\left(0\right)}={\hat{\theta }}_{0}$ where ${\hat{\theta }}_{0}$ is the vector of unpenalized estimates, that is, those which minimize $\ell_{0}(\theta )$ (defined in (1)).
• *Optimization.*
  – *Outer.* Minimize BIC(λ) with respect to λ using DEoptim, yielding $\lambda^{*}$ as defined in (8). Convergence occurs when $|\text{BIC}(\lambda^{\left(r+1\right)})-\text{BIC}(\lambda^{\left(r\right)})|$ is below a prespecified threshold. (Here, $\lambda^{\left(r\right)}$ is the best λ value found at step r of the DEoptim algorithm.)
  – *Inner.* For a given value of λ , maximize ℓ(θ) by iteratively re-solving the system of equations given in (5) starting from the initial value, $\theta^{\left(0\right)}$ ; this yields $\hat{\theta }$ . Convergence occurs when $||\theta^{\left(m+1\right)}-\theta^{\left(m\right)}|{|}_{\infty }$ is below a prespecified threshold. (Here $||y|{|}_{\infty }=\max_{j}|y_{j}|$ is the infinity norm.)
• *Output.* The estimates ${\hat{\theta }}^{*}$ corresponding to $\lambda^{*}$ , are returned from the above procedure, and the corresponding standard errors are calculated by evaluating (6) at ${\hat{\theta }}^{*}$ .

## 4. Simulation studies

### 4.1. Setup

The performance of the proposed variable selection methods is evaluated through simulation studies. The failure time is simulated from a Weibull MPR model with

$$\begin{array}{rl} \log\;(\tau_{i}) & =x_{i}^{T}(-1.5,-1.0,0.0,0.0,0.0,0.0,0.0,-0.8,0.5,0.0,0.0{)}^{T}\\ \log\;(\gamma_{i}) & =z_{i}^{T}(0.5,0.4,0.0,0.0,0.0,0.4,-0.2,0.0,0.0,0.0,0.0{)}^{T} \end{array}$$

where $x_{i}=z_{i}=(1,x_{i1},\ldots ,x_{i10}{)}^{T}$ is a vector of correlated variables generated from an AR(1) process with a correlation coefficient ρ=0.5 . Each variable is marginally standard normal and the correlation between any two consecutive variables $x_{ij}$ and $x_{ik}$ is given by $\rho^{|j-k|}$ . The corresponding censored times were generated from a uniform distribution such that the censoring proportion was $p_{\text{cen}}=25\%$ . This setup was chosen so as to yield realistic survival data, where the true model is sparse and the covariates are correlated. The results for three different sample sizes ( n = 100, 500, and 1000) are presented here. For each scenario, we considered the LASSO, SCAD, and ALASSO penalties with a single tuning parameter or two tuning parameters (i.e., one for each of the two regression components). Each simulation scenario was replicated 1000 times.

### 4.2. Simulation results

The variable selection and estimation procedures described in Sections 2 and 3 are applied to the simulated data and the results are summarized and discussed here. A number of metrics are used to evaluate the performance of the variable selection procedures, namely the average number of true zero coefficients *correctly* set to zero (*C*), the average number of true non-zero coefficients *incorrectly* set to zero (IC), and the probability of choosing the true model (PT); for the oracle model, *C* = 7 and IC = 0. As a measure of prediction accuracy, we also consider the mean squared error (MSE), given by $\text{MSE}(\hat{\beta })=(\hat{\beta }-\beta {)}^{T}S_{\beta }(\hat{\beta }-\beta )$ and $\text{MSE}(\hat{\alpha })=(\hat{\alpha }-\alpha {)}^{T}S_{\alpha }(\hat{\alpha }-\alpha )$ , where $S_{\beta }$ and $S_{\alpha }$ , the simulated sample covariance matrices of the covariates, are computed for each simulation replicate .^47,48^ These metrics, averaged over simulation replicates for the scenarios with 25% censoring, are reported in Table 2.

**Table 2.** Selection results: Variable selection metrics averaged over 1000 simulation replicates.

		LASSO				SCAD				ALASSO			
$p_{\text{cen}}$ = 25%	n	C(7)	IC(0)	PT	MSE	C(7)	IC(0)	PT	MSE	C(7)	IC(0)	PT	MSE
One tuning parameter													
	100	5.38	0.18	0.13	0.35	6.37	0.11	0.53	0.28	6.21	0.08	0.42	0.22
Scale ( β )	500	5.63	0.00	0.25	0.08	6.96	0.00	0.97	0.02	6.87	0.00	0.88	0.03
	1000	5.88	0.00	0.34	0.05	7.00	0.00	1.00	0.01	6.96	0.00	0.96	0.01
	100	3.61	0.08	0.02	0.05	4.49	0.09	0.05	0.05	6.37	0.22	0.45	0.04
Shape ( α )	500	3.46	0.00	0.01	0.01	5.95	0.00	0.34	0.01	6.89	0.00	0.90	0.00
	1000	3.48	0.00	0.00	0.00	6.52	0.00	0.63	0.00	6.95	0.00	0.95	0.00
Two tuning parameters													
	100	4.88	0.10	0.11	0.30	6.28	0.11	0.56	0.28	6.29	0.10	0.46	0.23
Scale ( β )	500	5.18	0.00	0.17	0.07	6.89	0.00	0.95	0.02	6.88	0.00	0.90	0.02
	1000	5.42	0.00	0.21	0.04	6.95	0.00	0.98	0.01	6.96	0.00	0.96	0.01
	100	5.08	0.26	0.09	0.06	5.06	0.18	0.10	0.05	6.44	0.26	0.45	0.04
Shape ( α )	500	5.42	0.00	0.21	0.01	6.10	0.00	0.42	0.01	6.90	0.00	0.91	0.00
	1000	5.57	0.00	0.26	0.00	6.51	0.00	0.62	0.00	6.96	0.00	0.96	0.00

C: average correct zeros; IC: average incorrect zeros; PT: the probability of choosing the true model; MSE: the average mean squared error; LASSO: least absolute shrinkage and selection operator;SCAD: smoothly clipped absolute deviation; ALASSO: adaptive least absolute shrinkage and selection operator.

As the sample size increases, we see an improvement across all four metrics, for both the shape and the scale parameters and across all penalties. However, it is evident that the LASSO penalty does not set enough covariates equal to zero (i.e., it selects an overly complex model). While the LASSO with one tuning parameter outperforms the LASSO with two tuning parameters in the scale component, it has very poor performance in the shape component (and we can also confirm that the BIC values are much higher). In any case, the LASSO over-selects irrespective of whether it has one or two tuning parameters, leading to quite low PT values. SCAD performs better than the LASSO, but still over-selects somewhat in the shape component. The best overall performance comes from the ALASSO penalty, which, for the largest sample size, selects the true scale and shape covariates more than 90% of the time. Interestingly, the ALASSO performs well even with a single tuning parameter (but it does improve with two tuning parameters). In terms of the computation time, SCAD has been found to be slower than the LASSO and ALASSO penalties. Furthermore, the computation times for the cases with two tuning parameters are two to three times longer than those with one tuning parameter.

**Figure 3.**
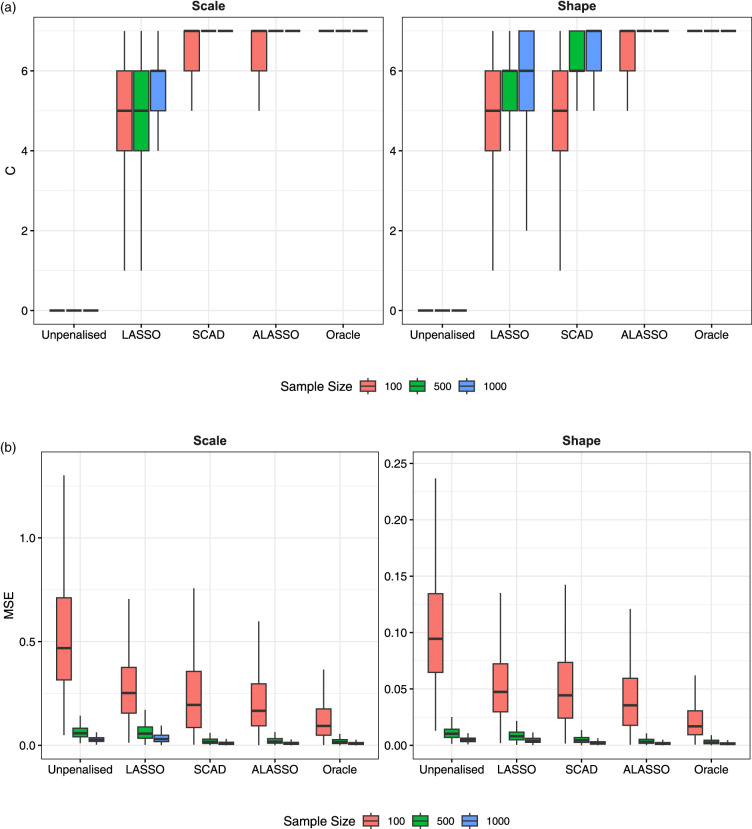
(a) True zero coefficients correctly set to zero (*C*) and (b) mean squared error (MSE) by model, distributional parameter and sample size across 1000 replicates for the models with two tuning parameters.

Figure 3 provides the boxplots of the C and MSE performance metrics over simulation replicates to account for variability in the results (for the penalties with two tuning parameters). Moreover, we additionally include the results for the full unpenalized and true oracle models, respectively, to act as worst-case and best-case benchmarks. It is clear that the C metric tends to be lower in the LASSO than SCAD and the ALASSO. The latter two are comparable in the scale component but the ALASSO outperforms SCAD in the shape, achieving the oracle value of *C* =7 when n≥500 with essentially no variation. In terms of MSE, we again see that SCAD and the ALASSO are similar (with lower values than LASSO). However, the ALASSO has slightly lower MSE values in the shape component and, as with the *C* metric, is very similar to the oracle model for n≥500 .

In addition to variable selection performance, we also consider parameter inference in terms of estimation bias, accuracy of the estimated standard error (SEE) computed using the sandwich formula, (6) compared to the true standard error (SE) compuated as the standard deviation over simulation replicates, and the empirical coverage probability (CP) of a nominal 95% confidence interval. The results for the ALASSO penalty (for the 25% censoring level) are presented in Table 3. Overall, we can see that the estimation bias and SEs reduce with the sample size and the CP values get closer to the nominal 95% level. However, at n=100 , the CP values are more than 10 percentage points lower than the nominal level in many cases. This is due to the parameters being overshrunk and the SEEs underestimating the SEs; this particularly impacts parameters which have smaller magnitudes (e.g. $\beta_{8}$ and $\alpha_{6}$ ). All results are improved by having two tuning parameters, and, indeed, for n=1000 , the CP values for most parameters are within 2 percentage points of the nominal value of 95%, and within 4 percentage points for $\beta_{8}$ and $\alpha_{6}$ . We defer LASSO and SCAD results to the Supplemental Material, where we find: the LASSO overshrinks parameters and underestimates the SEs; SCAD has lower bias, but underestimates the SEs such that CP values remain poor even at n=1000 (e.g., 20% for $\beta_{8}$ and 70% for $\alpha_{6}$ ).

**Table 3.** Inferential results: estimates, standard errors, and confidence intervals.

ALASSO													
		n = 100				n = 500				n = 1000			
$p_{\text{cen}}$ = 25%	θ	$\hat{\theta }$	SE	SEE	CP	$\hat{\theta }$	SE	SEE	CP	$\hat{\theta }$	SE	SEE	CP
One tuning parameter													
$\beta_{0}$	−1.50	−1.47	0.23	0.21	0.91	−1.48	0.09	0.09	0.93	−1.49	0.06	0.06	0.94
$\beta_{1}$	−1.00	−0.95	0.21	0.16	0.86	−0.98	0.07	0.07	0.92	−0.99	0.05	0.05	0.96
$\beta_{7}$	−0.80	−0.73	0.20	0.15	0.85	−0.77	0.06	0.06	0.92	−0.79	0.05	0.04	0.93
$\beta_{8}$	0.50	0.40	0.19	0.13	0.80	0.47	0.06	0.06	0.89	0.48	0.04	0.04	0.89
$\alpha_{0}$	0.50	0.52	0.11	0.09	0.91	0.50	0.04	0.04	0.95	0.50	0.03	0.03	0.94
$\alpha_{1}$	0.40	0.37	0.07	0.06	0.88	0.39	0.02	0.02	0.94	0.40	0.01	0.01	0.95
$\alpha_{5}$	0.40	0.35	0.08	0.06	0.78	0.39	0.03	0.02	0.91	0.39	0.02	0.02	0.91
$\alpha_{6}$	−0.20	−0.13	0.09	0.05	0.68	−0.18	0.03	0.02	0.87	−0.19	0.02	0.02	0.90
Two tuning parameters													
$\beta_{0}$	−1.50	−1.49	0.24	0.21	0.92	−1.49	0.09	0.09	0.94	−1.49	0.06	0.06	0.95
$\beta_{1}$	−1.00	−0.97	0.21	0.16	0.87	−0.99	0.07	0.07	0.93	−0.99	0.05	0.05	0.96
$\beta_{7}$	−0.80	−0.75	0.21	0.15	0.85	−0.78	0.06	0.06	0.94	−0.79	0.05	0.04	0.93
$\beta_{8}$	0.50	0.41	0.20	0.12	0.80	0.47	0.06	0.06	0.91	0.49	0.04	0.04	0.91
$\alpha_{0}$	0.50	0.53	0.11	0.09	0.90	0.50	0.04	0.04	0.95	0.50	0.03	0.03	0.94
$\alpha_{1}$	0.40	0.37	0.07	0.05	0.87	0.40	0.02	0.02	0.95	0.40	0.01	0.01	0.95
$\alpha_{5}$	0.40	0.35	0.09	0.06	0.77	0.39	0.03	0.02	0.92	0.39	0.02	0.02	0.92
$\alpha_{6}$	−0.20	−0.14	0.10	0.05	0.70	−0.19	0.03	0.02	0.88	−0.19	0.02	0.02	0.91

SE: standard deviation of estimates over 1000 replications; SEE: average of estimated standard errors over 1000 replications; CP: the empirical coverage probability of a nominal 95% confidence interval;ALASSO: adaptive least absolute shrinkage and selection operator.

**Figure 4.**
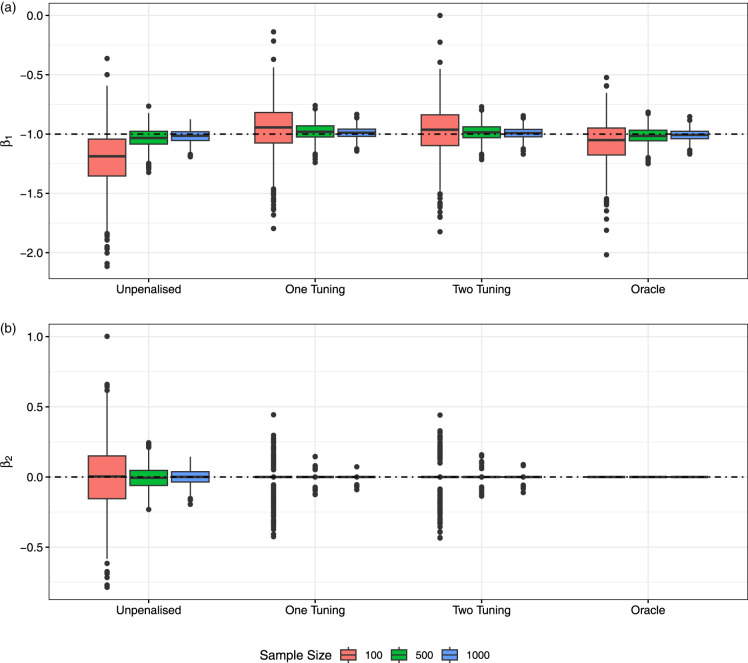
Coefficient estimates from the models with adaptive least absolute shrinkage and selection operator (ALASSO) penalties by sample size and across the 1000 replicates: (a) $\beta_{1}$ coefficient estimates and (b) $\beta_{2}$ coefficient estimates (the dashed line represents the true coefficient value).

**Figure 5.**
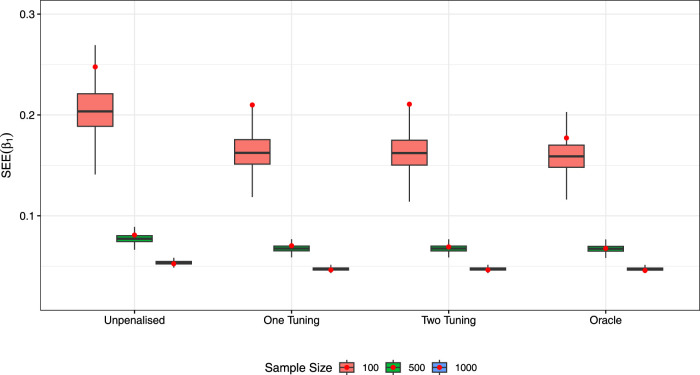
Boxplots of SEEs for $\beta_{1}$ along with SEs (dot). SEEs: estimated standard errors; SEs: standard errors.

Figure 4 displays the boxplots of estimates of $\beta_{1}$ (non-zero coefficient) and $\beta_{2}$ (zero coefficient) for the ALASSO over simulation replicates; for comparison, the full unpenalized and true oracle models are shown. It is clear that estimates from the ALASSO penalty (both one or two tuning parameters) converge to those of the oracle model, and, for n≥500 , we see that the zero coefficient is correctly set to zero in all but a small few outlying cases. The boxplots of the SEEs for $\beta_{1}$ are also shown in Figure 5, where we again see convergence to the oracle model (but note that SEs are underestimated for n=100 ). Similar boxplots for other parameters are shown in the Supplemental Material.

We have also tested all approaches at the higher censoring proportion of 50% (see Supplemental Material), where performance decreases across all metrics (e.g., reduced selection performance, increased bias, and variability), especially at smaller sample sizes. However, at n=1000 , the two-tuning-parameter ALASSO performs quite favorably, for example, PT >90 % and CP ∈[88%,96%] . We have further tested the ALASSO in additional simulation scenarios where we increased the correlation amongst covariates (from ρ=0.5 to ρ=0.8 ) and number of covariates (from 10 to 20), and decreased the proportion of non-zero effects (from 30% to 10%). Again, these can be found in the Supplemental Material, and, in all cases, the performance of the ALASSO is very favorable and broadly similar to the results already discussed.

## 5. Lung cancer study

Here we consider data from an observational lung cancer study which was collected by Wilkinson^49^ (see also Burke and MacKenzie^4^). This study includes all individuals, of all ages, diagnosed with lung cancer in Northern Ireland during the one-year period 1 October 1991 to 30 September 1992. Only cases of primary lung cancer were included. The date of diagnosis was taken to be the time origin for an individual and the end point was the earlier of the occurrence of death or the study end date, which was on 30 May 1993. Individuals who were still alive on the study end date were taken to have censored survival times. Individuals who died from another cause or who dropped out of the study were also censored. The final dataset included 855 patients, of which there were 673 deaths and 182 censored times. Besides the survival time and the censoring indicator, a number of other variables were recorded for each of the patients enrolled in the study (reference categories are listed first): age group (< 40-, 50-, 60-, 70-, and > 80), sex (female and male), treatment group (palliative, surgery, chemotherapy, radiotherapy, chemotherapy, and radiotherapy), WHO status (normal activity, light work, unable to work, >50% walking, and bed/chair bound), cancer cell type (squamous cell, small cell, adenocarcinoma, and other), serum sodium level ( ≥136mmol/L , <136mmol/L , missing), serum albumen level ( ≥35g/L , <35g/L , missing), metastases (no, yes, and unknown), and smoking status (non-smoker, current smoker, ex-smoker, and\break missing).

### 5.1. Adequacy of Weibull

**Figure 6.**
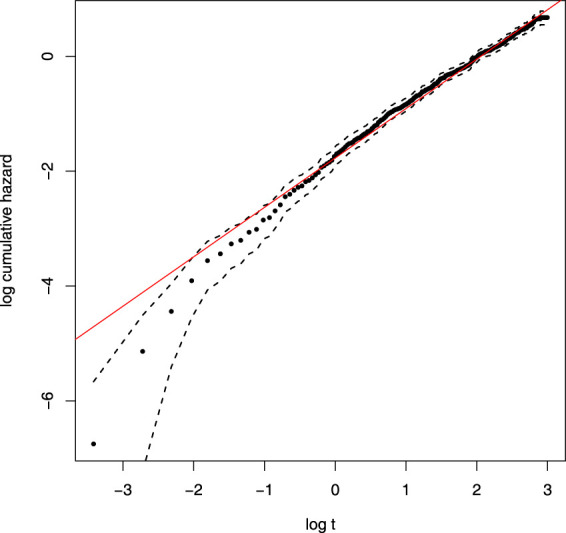
Weibull model check. Here $\hat{H}(t)$ , along with the 95% confidence intervals, come from the Kaplan–Meier estimator.

Before considering covariates and variable selection, we first carry out an initial check that a baseline Weibull distribution is appropriate for the lung cancer data. The cumulative hazard function for the Weibull model is given by $H(t)=\int_{0}^{t}h(u)du=\tau t^{\gamma }$ , and, hence, logH(t)=logτ+γlogt . Therefore, given an estimate $\hat{H}(t)$ , a plot of $\log\;\hat{H}(t)$ against logt should produce a straight line. This standard Weibull model check is shown in Figure 6, and, despite a slight deficiency for very small survival times, it appears that the Weibull model is reasonable.

### 5.2. Variable selection results

The variable selection results for the different penalties are summarized in Table 4. In line with the results of the simulation study, the LASSO penalty selects the most complex model and the ALASSO penalty selects the least complex. Both ALASSO penalties (one and two tuning parameter cases) are in agreement on the non-importance of sex and smoking status, and although age group is selected in the scale in the case with one tuning parameter, it is not significant. Interestingly, the two tuning parameter ALASSO selects the same set of covariates as identified by Burke and MacKenzie^4^ using a BIC stepwise procedure (albeit they additionally selected treatment in the shape). We also see that, in the two tuning parameters cases, the scale tuning parameter is smaller than that of the one tuning parameter case, while the shape tuning parameter is larger. This suggests that the single penalty over-penalizes the scale coefficients and under-penalizes the shape; this is also evident from the scale and shape degrees of freedom. Interestingly, the one tuning parameter ALASSO converges in less than half the time of the two tuning parameter ALASSO, and achieves similar results. We expect this based on our simulation studies, and also expect the results of the two tuning parameter case to be marginally better (albeit it takes longer to converge).

**Table 4.** Summary of penalized models (lung cancer data).

	One tuning parameter			Two tuning parameters		
	LASSO	SCAD	ALASSO	LASSO	SCAD	ALASSO
Treatment	β , α	β , α	β , α	β , α	β , α	β
Age group	α	α	β	α	α	–
WHO status	β , α	β , α	β	β , α	β , α	β
Sex	α	–	–	–	–	–
Smoking status	α	α	–	β	β	–
Cell type	β , α	β , α	β	β	β , α	β
Metastases	β , α	β , α	β	β	β	β
Sodium	β , α	β , α	β	β	β	β
Albumen	β , α	β , α	β	β , α	β , α	β
Tuning parameter(s)	0.026	0.041	0.015	0.014	0.024	0.004
				0.080	0.074	0.045
Degrees of freedom	32.5	27.1	15.5	25.6	24.6	15.2
Scale degrees of freedom	14.2	12.0	13.2	18.3	17.1	14.2
Shape degrees of freedom	18.3	15.0	2.4	7.4	7.6	1.0

β = “selected in scale,” α = “selected in shape,” and those which are non-significant (at the 5% level) are shown in gray. LASSO: least absolute shrinkage and selection operator; SCAD: smoothly clipped absolute deviation; ALASSO: adaptive least absolute shrinkage and selection operator; WHO: World Health Organization.

Table 5 displays the estimated coefficients for both ALASSO penalties along with the unpenalized coefficients (we focus on the ALASSO due to its superior performance in our simulation studies, but similar tables for LASSO and SCAD can be found in the Supplemental Material). Note that the scale coefficients characterize the overall scale of the hazard (a positive value indicates an increase relative to the reference category), while the shape coefficients characterize its time evolution (a positive value indicates a hazard which increases over time relative to the reference category). We clearly see the similarity of the coefficient values for both the one and two tuning parameter ALASSO penalties, and, furthermore, that the selected variables are broadly in line with those which are statistically significant in the unpenalized model. Focusing on the results of the two tuning parameter case we find that all treatments (apart from chemotherapy) have a negative scale coefficient suggesting that treatment reduces hazard (relative to palliative care); however, worse WHO status, small cancer cell type, presence of metasteses, and reduced sodium and albumen levels increase the hazard; lastly, sex, age group, and smoking status have no significant effect on the hazard. Since no variable appears in the shape component (i.e. all shape coefficients are set to zero), the selected model is a PH model, and exponentiating the scale coefficients yields the hazard ratios, for example, the surgery hazard ratio is exp(−0.98)=0.375 so that the risk of death is approximately 37.5% that of a patient receiving palliative care.

**Table 5.** Coefficients estimates and standard errors for the ALASSO penalties (lung cancer data).

		Scale			Shape		
Covariate		No penalty	One tuning	Two tuning	No penalty	One tuning	Two tuning
Intercept		−3.38 (**0.66**)	−3.12 (**0.17**)	−3.15 (**0.17**)	−0.16 (0.22)	0.04 (0.03)	0.04 (0.03)
Treatment	Surgery	−1.69 (**0.83**)	−0.89 (**0.21**)	−0.98 (**0.22**)	0.11 (0.21)	0.00 (0.00)	0.00 (0.00)
	Chemotherapy	−0.33 (0.37)	0.00 (0.00)	0.00 (0.00)	−0.03 (0.15)	0.00 (0.00)	0.00 (0.00)
	Radiotherapy	−0.85 (**0.21**)	−0.16 (0.10)	−0.21 (**0.10**)	**0.22** (**0.08**)	0.00 (0.00)	0.00 (0.00)
	Chemo. and radio.	−3.83 (**0.98**)	−2.30 (**0.89**)	−0.63 (**0.22**)	**0.77** (**0.20**)	**0.51** (**0.21**)	0.00 (0.00)
Age group	50−	−0.90 (**0.43**)	0.00 (0.00)	0.00 (0.00)	**0.39** (**0.16**)	0.00 (0.00)	0.00 (0.00)
	60−	−0.94 (**0.39**)	0.00 (0.00)	0.00 (0.00)	**0.40** (**0.15**)	0.00 (0.00)	0.00 (0.00)
	70−	−0.77 (0.39)	0.02 (0.08)	0.00 (0.00)	**0.31** (**0.15**)	0.00 (0.00)	0.00 (0.00)
	>80	−0.78 (0.42)	0.00 (0.00)	0.00 (0.00)	0.31 (0.17)	0.00 (0.00)	0.00 (0.00)
WHO status	Light work	−0.02 (0.45)	0.00 (0.00)	0.00 (0.00)	0.02 (0.12)	0.00 (0.00)	0.00 (0.00)
	Unable to work	0.84 (0.43)	**0.41** (**0.10**)	0.44 (**0.10**)	−0.10 (0.13)	0.00 (0.00)	0.00 (0.00)
	>50% walking	**1.31** (**0.44**)	**0.99** (**0.11**)	**0.97** (**0.11**)	−0.13 (0.14)	0.00 (0.00)	0.00 (0.00)
	Bed/chair bound	**1.78** (**0.50**)	**1.28** (**0.28**)	**1.54** (**0.25**)	−0.03 (0.20)	0.00 (0.00)	0.00 (0.00)
Sex	Male	0.03 (0.14)	0.00 (0.00)	0.00 (0.00)	−0.03 (0.05)	0.00 (0.00)	0.00 (0.00)
Smoking status	Current smoker	0.10 (0.22)	0.00 (0.00)	0.00 (0.00)	0.15 (0.08)	0.00 (0.00)	0.00 (0.00)
	Ex-smoker	−0.05 (0.23)	0.00 (0.00)	0.00 (0.00)	0.17 (0.09)	0.00 (0.00)	0.00 (0.00)
	Missing	0.29 (0.40)	0.00 (0.00)	0.00 (0.00)	0.00 (0.00)	0.00 (0.00)	0.00 (0.00)
Cell type	Small cell	**0.83** (**0.26**)	**0.31** (**0.12**)	**0.43** (**0.13**)	−0.05 (0.10)	0.00 (0.00)	0.00 (0.00)
	Adenocarcinoma	0.28 (0.28)	0.00 (0.00)	0.00 (0.00)	0.03 (0.10)	0.00 (0.00)	0.00 (0.00)
	Other	0.32 (0.20)	0.00 (0.00)	0.09 (0.09)	−0.04 (0.07)	0.00 (0.00)	0.00 (0.00)
Metastases	Yes	**1.35** (**0.28**)	**0.89** (**0.12**)	**0.84** (**0.12**)	−0.19 (**0.08**)	0.00 (0.00)	0.00 (0.00)
	Unknown	**0.83** (**0.30**)	**0.53** (**0.13**)	**0.41** (**0.13**)	−0.14 (0.09)	0.00 (0.00)	0.00 (0.00)
Sodium level	<136mmol/L	**0.33** (**0.14**)	0.14 (0.08)	**0.24** (**0.08**)	−0.01 (0.05)	0.00 (0.00)	0.00 (0.00)
	Missing	−0.77 (0.45)	0.00 (0.00)	0.00 (0.00)	**0.32** (**0.16**)	0.00 (0.00)	0.00 (0.00)
Albumen level	<35g/L	**0.65** (**0.16**)	**0.36** (**0.09**)	**0.37** (**0.09**)	−0.10 (0.06)	0.00 (0.00)	0.00 (0.00)
	Missing	**0.59** (**0.28**)	0.00 (0.00)	**0.27** (**0.14**)	0.09 (0.15)	0.00 (0.00)	0.00 (0.00)

ALASSO: adaptive least absolute shrinkage and selection operator; WHO: World Health Organization. Bold indicates statistically significant at the 5% level.

## 6. Discussion

The MPR approach results in flexible models which extend standard models, but the presence of multiple regression components means that variable selection is necessarily more challenging than in standard settings where there is only a single regression component. In this article, we have proposed a penalized variable selection procedure for the simultaneous selection of variables in the scale and shape parameters of a Weibull MPR model in the survival analysis setting. The favorable performance of these methods was examined using simulation studies, and an analysis of lung cancer data was presented. While we have considered the Weibull model example in this article, the proposed variable selection procedures can be applied straightforwardly to other MPR survival models by adapting the likelihood function.

Given that we model different distributional parameters (a scale and a shape parameter), there is no reason to assume that variable selection can be achieved with a single penalty applied to both regression components; hence, we also investigated the need for a separate tuning parameter for each regression component. We have found that the ALASSO performs very well in terms of identifying the true subset of covariates and coverage of calculated confidence intervals. This is true even with a single tuning parameter, however the results are improved when there are two tuning parameters (albeit this is more computationally intensive). On the other hand, SCAD does not perform well in the MPR setting, selecting an overly complex model and with poor confidence interval coverage for shape parameters.

## Supplemental Material

sj-pdf-1-smm-10.1177_09622802231203322 - Supplemental material for Penalized variable selection in multi-parameter regression survival modeling Click here for additional data file. Supplemental material, sj-pdf-1-smm-10.1177_09622802231203322 for Penalized variable selection in multi-parameter regression survival modeling by Fatima-Zahra Jaouimaa, Il Do Ha and Kevin Burke in Statistical Methods in Medical Research
