# Supplementary material for: Penalized variable selection in multi-parameter regression survival modeling
Source: Stat Methods Med Res. 2023 Oct 12;32(12):2455–71. doi: 10.1177/09622802231203322 (PMC10710000; doi:10.1177/09622802231203322)
Supplement: sj-pdf-1-smm-10.1177_09622802231203322 - Supplemental material for Penalized variable selection in multi-parameter regression survival modeling [file sj-pdf-1-smm-10.1177_09622802231203322.pdf]

Journal Title
XX(X):1–??

©The Author(s) 2019
Reprints and permission:
sagepub.co.uk/journalsPermissions.nav

DOI: 10.1177/ToBeAssigned
www.sagepub.com/
SAGE

# Supplementary Material for "Penalized Variable Selection in Multi-Parameter Regression Survival Modelling"

**Fatima-Zahra Jaouimaa[1], Il Do Ha[2] and Kevin Burke[1]**

**Abstract**

This Supplementary Material file contains the following.

**Section 1** A plot of the BIC objective with two tuning parameters.

**Section 2** Additional inferential results from the simulation study (as only the ALASSO was covered in the main paper).

**Section 3** Simulation results with 50% censoring.

**Section 4** Further simulation scenarios for the ALASSO approach where the correlation amongst covariates is increased (from $\rho = 0.5$ to $\rho = 0.8$), the number of covariates is increased (from 10 to 20), and the proportion of non-zero effects is decreased (from 30% to 10%).

**Section 5** An exploration of model-based (Bayesian) optimization and grid search for tuning parameter selection.

**Section 6** Tables of estimated coefficients and standard errors for all methods applied to the lung cancer data from the main paper.

[1]Department of Mathematics and Statistics, University of Limerick, Ireland
[2]Department of Statistics, Pukyong National University, Korea

**Corresponding author:**
Kevin Burke, Department of Mathematics and Statistics, University of Limerick, Ireland.
Email: kevin.burke@ul.ie

## 1 BIC function with two tuning parameters

Figure 1 is equivalent to Figure 2 from the main paper, but in the situation where there are two tuning parameters. Due to the various crests and troughs, we have suggested the use of the `DEoptim` "global" optimizer.

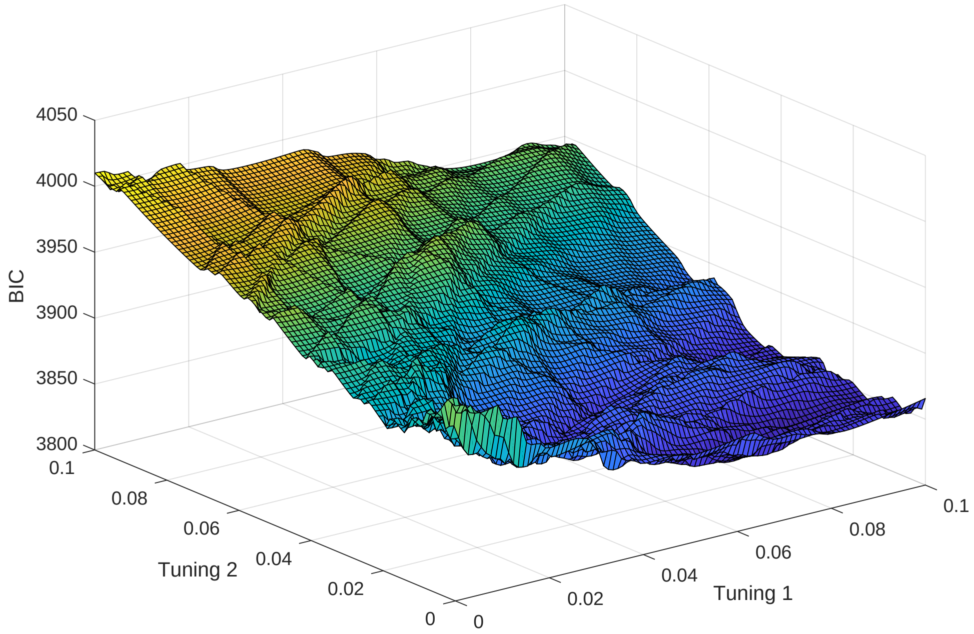


**Figure 1.** The BIC function evaluated at different tuning parameter values for the Weibull MPR model with two tuning parameter LASSO penalty for the lung cancer data analysed in Section 5 of the manuscript.

## 2 Additional inferential results

In the simulation study in the main paper, we focused on the inferential results for the ALASSO approach. For completeness, we include here the results for the LASSO (Table 1), SCAD (Table 2), and the unpenalised full and oracle models (Table 3).

**Table 1.** LASSO inferential results: estimates, standard errors, and confidence intervals.

| LASSO | | | | | | | | | | | | | |
|---|---|---|---|---|---|---|---|---|---|---|---|---|---|
| One Tuning Parameter | | | | | | | | | | | | | |
| $p_{\text{cen}}$ = 25% | | $n$ = 100 | | | | $n$ = 500 | | | | $n$ = 1000 | | | |
| | $\theta$ | $\hat{\theta}$ | SE | SEE | CP | $\hat{\theta}$ | SE | SEE | CP | $\hat{\theta}$ | SE | SEE | CP |
| $\beta_0$ | −1.50 | −1.34 | 0.24 | 0.20 | 0.80 | −1.38 | 0.09 | 0.09 | 0.70 | −1.40 | 0.07 | 0.06 | 0.61 |
| $\beta_1$ | −1.00 | −0.81 | 0.23 | 0.17 | 0.71 | −0.89 | 0.08 | 0.07 | 0.62 | −0.91 | 0.05 | 0.05 | 0.54 |
| $\beta_7$ | −0.80 | −0.54 | 0.20 | 0.16 | 0.61 | −0.66 | 0.08 | 0.07 | 0.49 | −0.70 | 0.06 | 0.05 | 0.43 |
| $\beta_8$ | 0.50 | 0.27 | 0.18 | 0.13 | 0.63 | 0.37 | 0.07 | 0.07 | 0.49 | 0.40 | 0.05 | 0.05 | 0.43 |
| $\alpha_0$ | 0.50 | 0.47 | 0.12 | 0.10 | 0.88 | 0.46 | 0.04 | 0.04 | 0.83 | 0.47 | 0.03 | 0.03 | 0.76 |
| $\alpha_1$ | 0.40 | 0.34 | 0.08 | 0.07 | 0.85 | 0.38 | 0.03 | 0.03 | 0.88 | 0.38 | 0.02 | 0.02 | 0.85 |
| $\alpha_5$ | 0.40 | 0.35 | 0.08 | 0.07 | 0.88 | 0.38 | 0.03 | 0.03 | 0.91 | 0.39 | 0.02 | 0.02 | 0.90 |
| $\alpha_6$ | −0.20 | −0.14 | 0.08 | 0.07 | 0.85 | −0.18 | 0.03 | 0.03 | 0.89 | −0.19 | 0.02 | 0.02 | 0.90 |
| Two Tuning Parameters | | | | | | | | | | | | | |
| $p_{\text{cen}}$ = 25% | | $n$ = 100 | | | | $n$ = 500 | | | | $n$ = 1000 | | | |
| | $\theta$ | $\hat{\theta}$ | SE | SEE | CP | $\hat{\theta}$ | SE | SEE | CP | $\hat{\theta}$ | SE | SEE | CP |
| $\beta_0$ | −1.50 | −1.38 | 0.23 | 0.20 | 0.84 | −1.40 | 0.09 | 0.09 | 0.76 | −1.42 | 0.07 | 0.06 | 0.71 |
| $\beta_1$ | −1.00 | −0.84 | 0.21 | 0.17 | 0.76 | −0.89 | 0.08 | 0.07 | 0.66 | −0.92 | 0.05 | 0.05 | 0.59 |
| $\beta_7$ | −0.80 | −0.63 | 0.20 | 0.16 | 0.75 | −0.70 | 0.07 | 0.07 | 0.65 | −0.72 | 0.05 | 0.05 | 0.60 |
| $\beta_8$ | 0.50 | 0.34 | 0.18 | 0.13 | 0.75 | 0.41 | 0.07 | 0.06 | 0.62 | 0.43 | 0.05 | 0.04 | 0.57 |
| $\alpha_0$ | 0.50 | 0.50 | 0.11 | 0.10 | 0.90 | 0.47 | 0.04 | 0.04 | 0.88 | 0.48 | 0.03 | 0.03 | 0.85 |
| $\alpha_1$ | 0.40 | 0.33 | 0.08 | 0.06 | 0.80 | 0.37 | 0.03 | 0.02 | 0.79 | 0.38 | 0.02 | 0.02 | 0.75 |
| $\alpha_5$ | 0.40 | 0.32 | 0.09 | 0.07 | 0.72 | 0.36 | 0.03 | 0.03 | 0.72 | 0.37 | 0.02 | 0.02 | 0.66 |
| $\alpha_6$ | −0.20 | −0.11 | 0.09 | 0.05 | 0.68 | −0.16 | 0.03 | 0.03 | 0.69 | −0.17 | 0.02 | 0.02 | 0.64 |

SE, standard deviation of estimates over 1000 replications; SEE, average of estimated standard errors over 1000 replications; CP, the empirical coverage probability of a nominal 95% confidence interval.

**Table 2.** SCAD inferential results: estimates, standard errors, and confidence intervals.

| SCAD | | | | | | | | | | | | | |
|---|---|---|---|---|---|---|---|---|---|---|---|---|---|
| One Tuning Parameter | | | | | | | | | | | | | |
| $p_{\text{cen}}$ = 25% | | $n$ = 100 | | | | $n$ = 500 | | | | $n$ = 1000 | | | |
| | $\theta$ | $\hat{\theta}$ | SE | SEE | CP | $\hat{\theta}$ | SE | SEE | CP | $\hat{\theta}$ | SE | SEE | CP |
| $\beta_0$ | −1.50 | −1.62 | 0.25 | 0.22 | 0.91 | −1.52 | 0.09 | 0.09 | 0.95 | −1.51 | 0.06 | 0.06 | 0.94 |
| $\beta_1$ | −1.00 | −1.08 | 0.21 | 0.17 | 0.89 | −1.01 | 0.07 | 0.07 | 0.94 | −1.01 | 0.05 | 0.05 | 0.95 |
| $\beta_7$ | −0.80 | −0.85 | 0.23 | 0.16 | 0.86 | −0.82 | 0.06 | 0.06 | 0.94 | −0.81 | 0.05 | 0.04 | 0.93 |
| $\beta_8$ | 0.50 | 0.50 | 0.25 | 0.12 | 0.62 | 0.51 | 0.06 | 0.05 | 0.78 | 0.50 | 0.04 | 0.04 | 0.87 |
| $\alpha_0$ | 0.50 | 0.59 | 0.11 | 0.10 | 0.80 | 0.52 | 0.04 | 0.04 | 0.92 | 0.51 | 0.03 | 0.03 | 0.92 |
| $\alpha_1$ | 0.40 | 0.39 | 0.07 | 0.06 | 0.84 | 0.40 | 0.02 | 0.02 | 0.82 | 0.40 | 0.02 | 0.01 | 0.80 |
| $\alpha_5$ | 0.40 | 0.37 | 0.09 | 0.06 | 0.77 | 0.38 | 0.03 | 0.02 | 0.70 | 0.39 | 0.02 | 0.01 | 0.69 |
| $\alpha_6$ | −0.20 | −0.15 | 0.09 | 0.06 | 0.66 | −0.17 | 0.03 | 0.02 | 0.77 | −0.18 | 0.02 | 0.02 | 0.74 |
| Two Tuning Parameters | | | | | | | | | | | | | |
| $p_{\text{cen}}$ = 25% | | $n$ = 100 | | | | $n$ = 500 | | | | $n$ = 1000 | | | |
| | $\theta$ | $\hat{\theta}$ | SE | SEE | CP | $\hat{\theta}$ | SE | SEE | CP | $\hat{\theta}$ | SE | SEE | CP |
| $\beta_0$ | −1.50 | −1.60 | 0.26 | 0.22 | 0.88 | −1.52 | 0.09 | 0.09 | 0.94 | −1.51 | 0.06 | 0.06 | 0.93 |
| $\beta_1$ | −1.00 | −1.07 | 0.21 | 0.18 | 0.88 | −1.01 | 0.07 | 0.07 | 0.94 | −1.01 | 0.05 | 0.05 | 0.95 |
| $\beta_7$ | −0.80 | −0.86 | 0.22 | 0.16 | 0.84 | −0.82 | 0.06 | 0.06 | 0.91 | −0.81 | 0.05 | 0.04 | 0.87 |
| $\beta_8$ | 0.50 | 0.52 | 0.24 | 0.08 | 0.39 | 0.50 | 0.06 | 0.01 | 0.23 | 0.50 | 0.04 | 0.01 | 0.21 |
| $\alpha_0$ | 0.50 | 0.58 | 0.11 | 0.09 | 0.80 | 0.52 | 0.04 | 0.04 | 0.92 | 0.51 | 0.03 | 0.03 | 0.91 |
| $\alpha_1$ | 0.40 | 0.38 | 0.08 | 0.06 | 0.82 | 0.40 | 0.02 | 0.02 | 0.80 | 0.40 | 0.01 | 0.01 | 0.78 |
| $\alpha_5$ | 0.40 | 0.35 | 0.09 | 0.06 | 0.69 | 0.38 | 0.03 | 0.02 | 0.66 | 0.39 | 0.02 | 0.01 | 0.63 |
| $\alpha_6$ | −0.20 | −0.13 | 0.09 | 0.05 | 0.54 | −0.17 | 0.03 | 0.02 | 0.67 | −0.18 | 0.02 | 0.02 | 0.72 |

SE, standard deviation of estimates over 1000 replications; SEE, average of estimated standard errors over 1000 replications; CP, the empirical coverage probability of a nominal 95% confidence interval.

**Table 3.** Unpenalised full and oracle model inferential results: estimates, standard errors, and confidence intervals.

| Unpenalised | | | | | | | | | | | | | |
|---|---|---|---|---|---|---|---|---|---|---|---|---|---|
| Full Model | | | | | | | | | | | | | |
| $p_{\text{cen}}$ = 25% | | $n$ = 100 | | | | $n$ = 500 | | | | $n$ = 1000 | | | |
| | $\theta$ | $\hat{\theta}$ | SE | SEE | CP | $\hat{\theta}$ | SE | SEE | CP | $\hat{\theta}$ | SE | SEE | CP |
| $\beta_0$ | −1.50 | −1.77 | 0.28 | 0.25 | 0.82 | −1.55 | 0.10 | 0.09 | 0.93 | −1.52 | 0.07 | 0.07 | 0.94 |
| $\beta_1$ | −1.00 | −1.20 | 0.25 | 0.21 | 0.83 | −1.03 | 0.08 | 0.08 | 0.92 | −1.02 | 0.05 | 0.05 | 0.94 |
| $\beta_7$ | −0.80 | −0.96 | 0.25 | 0.22 | 0.86 | −0.82 | 0.08 | 0.08 | 0.94 | −0.81 | 0.06 | 0.06 | 0.94 |
| $\beta_8$ | 0.50 | 0.61 | 0.24 | 0.21 | 0.90 | 0.51 | 0.08 | 0.08 | 0.93 | 0.51 | 0.06 | 0.05 | 0.94 |
| $\alpha_0$ | 0.50 | 0.66 | 0.11 | 0.10 | 0.62 | 0.53 | 0.04 | 0.04 | 0.89 | 0.52 | 0.03 | 0.03 | 0.92 |
| $\alpha_1$ | 0.40 | 0.41 | 0.08 | 0.07 | 0.90 | 0.40 | 0.03 | 0.03 | 0.94 | 0.40 | 0.02 | 0.02 | 0.94 |
| $\alpha_5$ | 0.40 | 0.42 | 0.10 | 0.09 | 0.90 | 0.40 | 0.04 | 0.03 | 0.94 | 0.40 | 0.02 | 0.02 | 0.95 |
| $\alpha_6$ | −0.20 | −0.21 | 0.10 | 0.08 | 0.88 | −0.20 | 0.03 | 0.03 | 0.93 | −0.20 | 0.02 | 0.02 | 0.94 |
| Oracle Model | | | | | | | | | | | | | |
| $p_{\text{cen}}$ = 25% | | $n$ = 100 | | | | $n$ = 500 | | | | $n$ = 1000 | | | |
| | $\theta$ | $\hat{\theta}$ | SE | SEE | CP | $\hat{\theta}$ | SE | SEE | CP | $\hat{\theta}$ | SE | SEE | CP |
| $\beta_0$ | −1.50 | −1.58 | 0.22 | 0.21 | 0.93 | −1.52 | 0.09 | 0.09 | 0.95 | −1.51 | 0.06 | 0.06 | 0.94 |
| $\beta_1$ | −1.00 | −1.07 | 0.18 | 0.16 | 0.91 | −1.01 | 0.07 | 0.07 | 0.94 | −1.01 | 0.05 | 0.05 | 0.96 |
| $\beta_7$ | −0.80 | −0.85 | 0.15 | 0.15 | 0.94 | −0.81 | 0.06 | 0.06 | 0.96 | −0.81 | 0.05 | 0.04 | 0.94 |
| $\beta_8$ | 0.50 | 0.53 | 0.14 | 0.13 | 0.95 | 0.51 | 0.06 | 0.06 | 0.96 | 0.50 | 0.04 | 0.04 | 0.94 |
| $\alpha_0$ | 0.50 | 0.55 | 0.10 | 0.09 | 0.90 | 0.51 | 0.04 | 0.04 | 0.94 | 0.51 | 0.03 | 0.03 | 0.94 |
| $\alpha_1$ | 0.40 | 0.40 | 0.05 | 0.05 | 0.94 | 0.40 | 0.02 | 0.02 | 0.95 | 0.40 | 0.01 | 0.01 | 0.95 |
| $\alpha_5$ | 0.40 | 0.40 | 0.06 | 0.06 | 0.93 | 0.40 | 0.02 | 0.02 | 0.95 | 0.40 | 0.02 | 0.02 | 0.94 |
| $\alpha_6$ | −0.20 | −0.20 | 0.06 | 0.06 | 0.93 | −0.20 | 0.02 | 0.02 | 0.93 | −0.20 | 0.02 | 0.02 | 0.95 |

SE, standard deviation of estimates over 1000 replications; SEE, average of estimated standard errors over 200 replications; CP, the empirical coverage probability of a nominal 95% confidence interval.

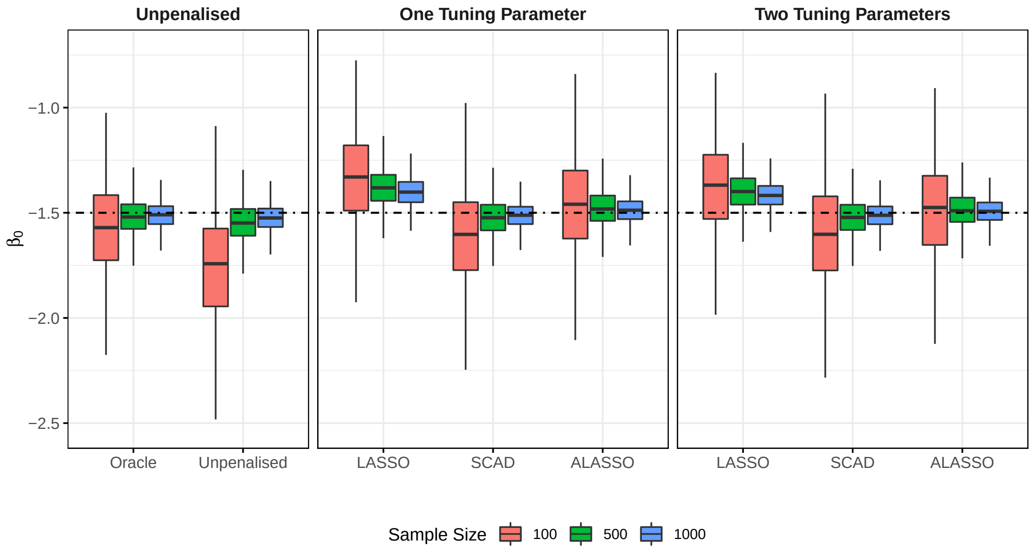


**(a)**

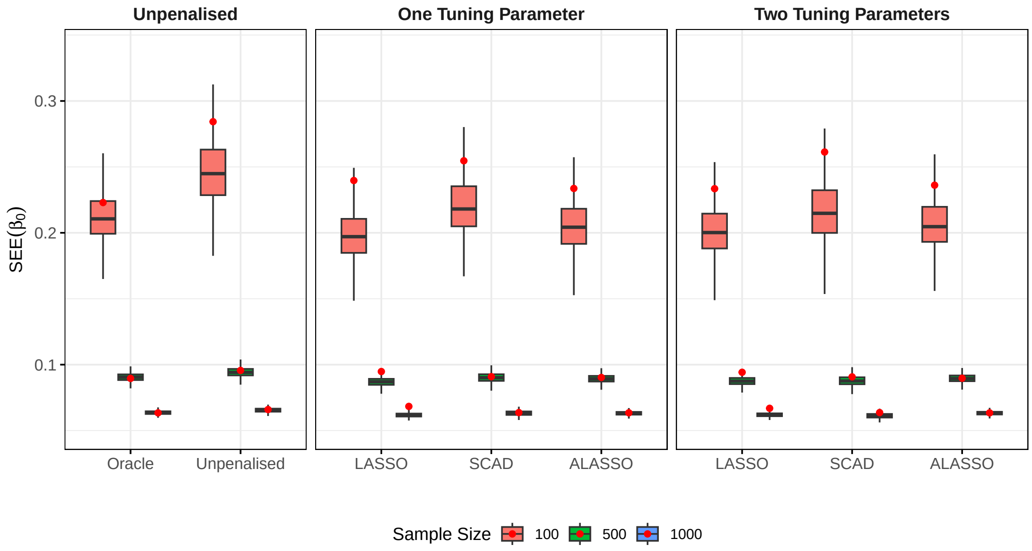


**(b)**

**Figure 2.** (a) $\beta_0$ coefficient estimates and (b) the corresponding estimated standard error (SEE) and standard deviation (SE) by model and sample size across 1000 replicates (the dashed line across the plots in panel (a) represents the true coefficient value and the dot in panel (b) is for the SE).

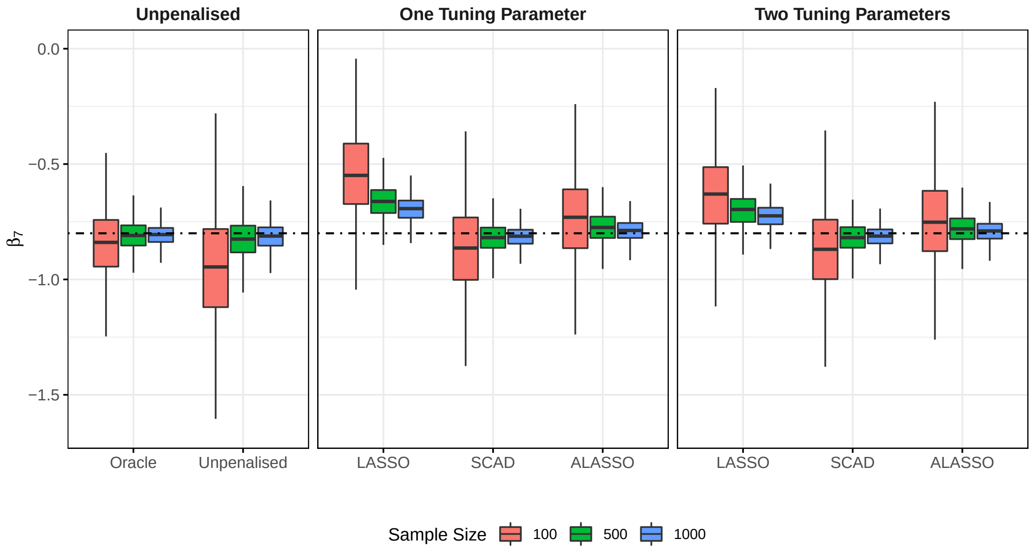


**(a)**

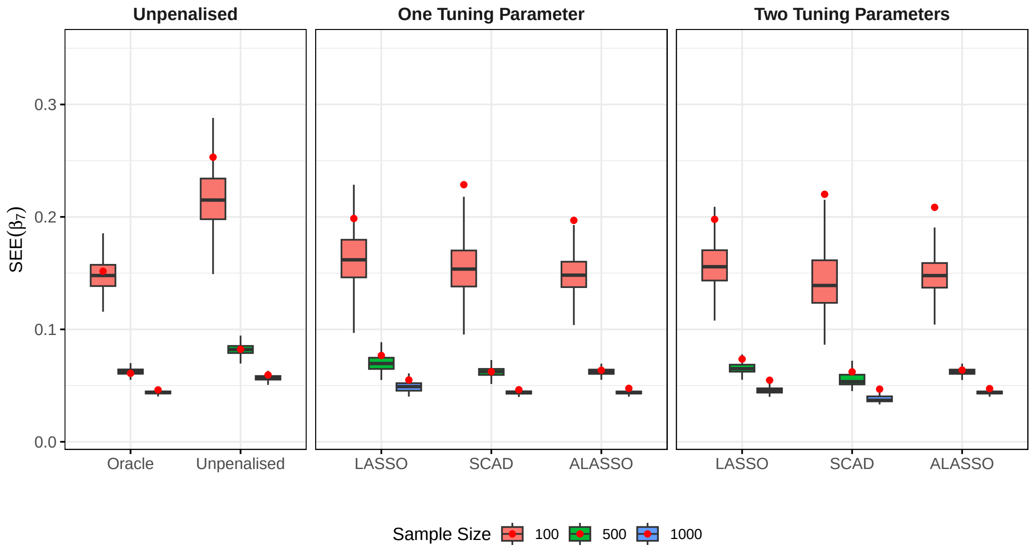


**(b)**

**Figure 3.** (a) $\beta_7$ coefficient estimates and (b) the corresponding estimated standard error (SEE) and standard deviation (SE) by model and sample size across 1000 replicates (the dashed line across the plots in panel (a) represents the true coefficient value and the dot in panel (b) is for the SE).

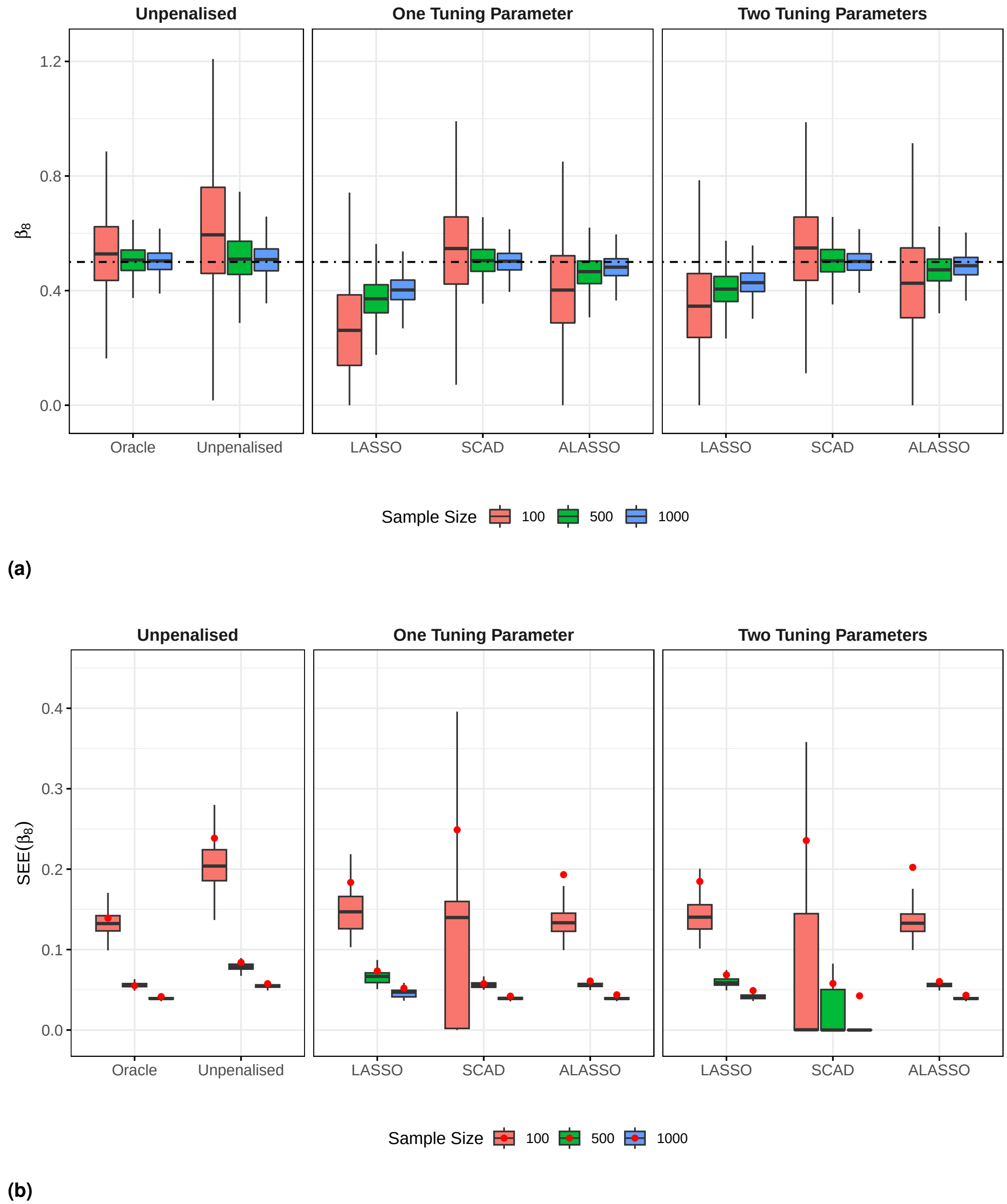


**Figure 4.** (a) $\beta_8$ coefficient estimates and (b) the corresponding estimated standard error (SEE) and standard deviation (SE) by model and sample size across 1000 replicates (the dashed line across the plots in panel (a) represents the true coefficient value and the dot in panel (b) is for the SE).

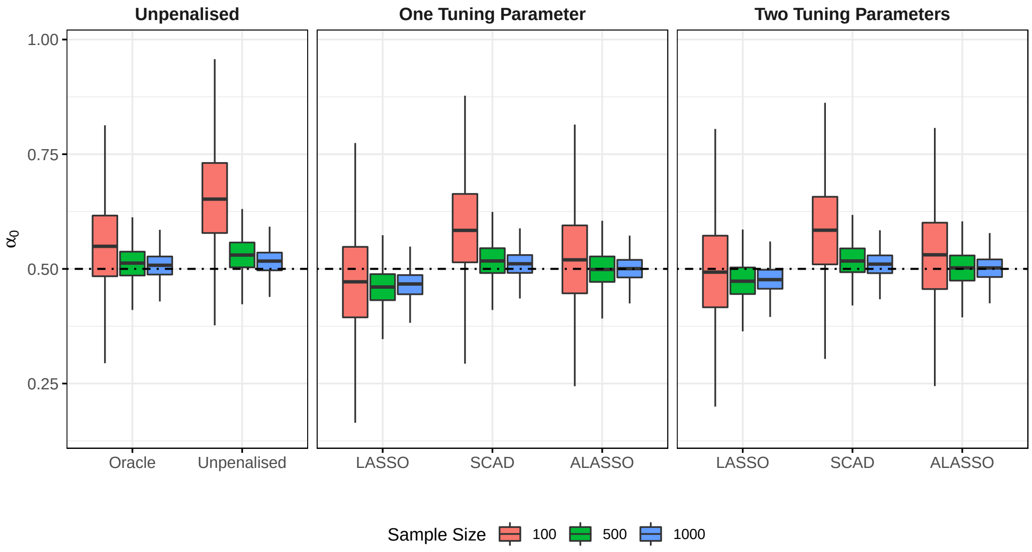


**(a)**

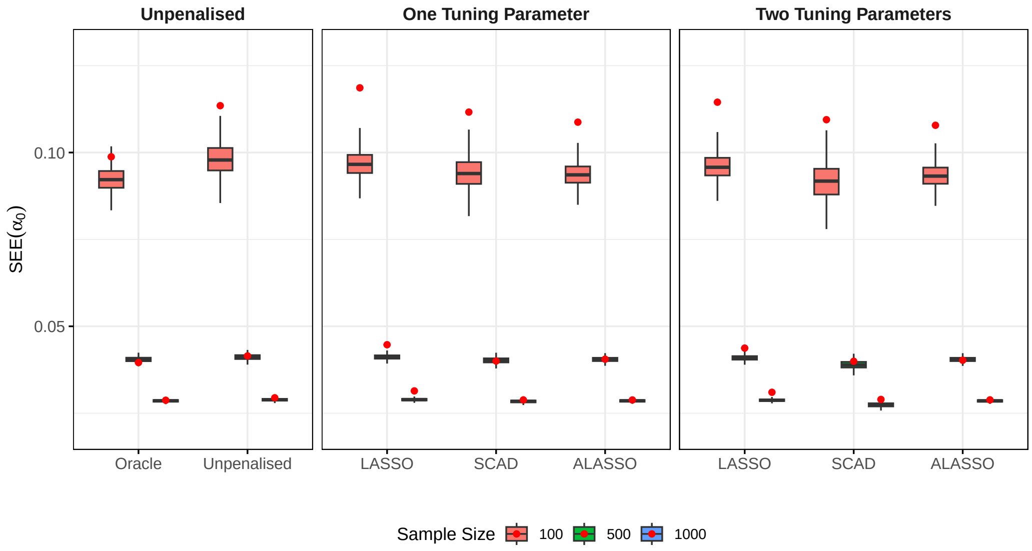


**(b)**

**Figure 5.** (a) $\alpha_0$ coefficient estimates and (b) the corresponding estimated standard error (SEE) and standard deviation (SE) by model and sample size across 1000 replicates (the dashed line across the plots in panel (a) represents the true coefficient value and the dot in panel (b) is for the SE).

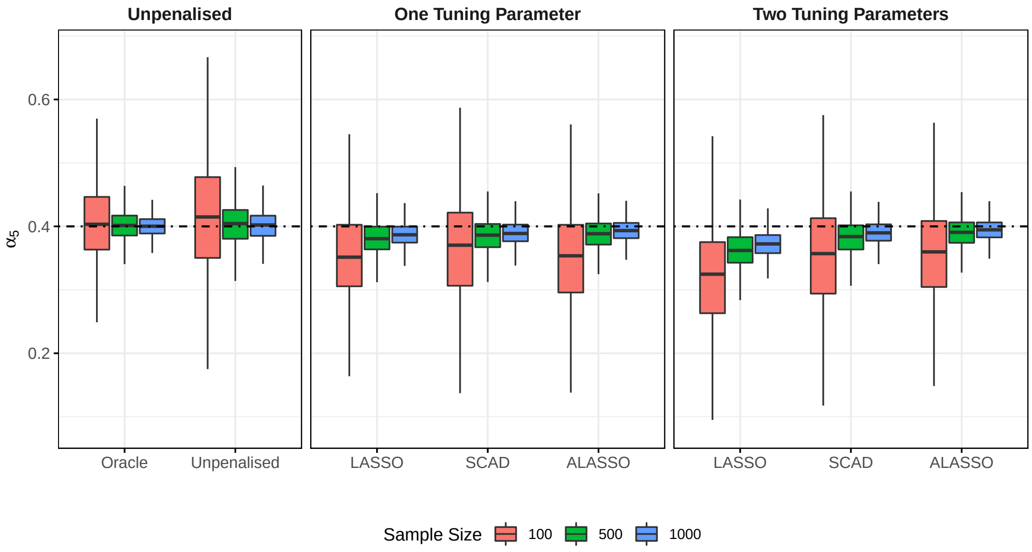


**(a)**

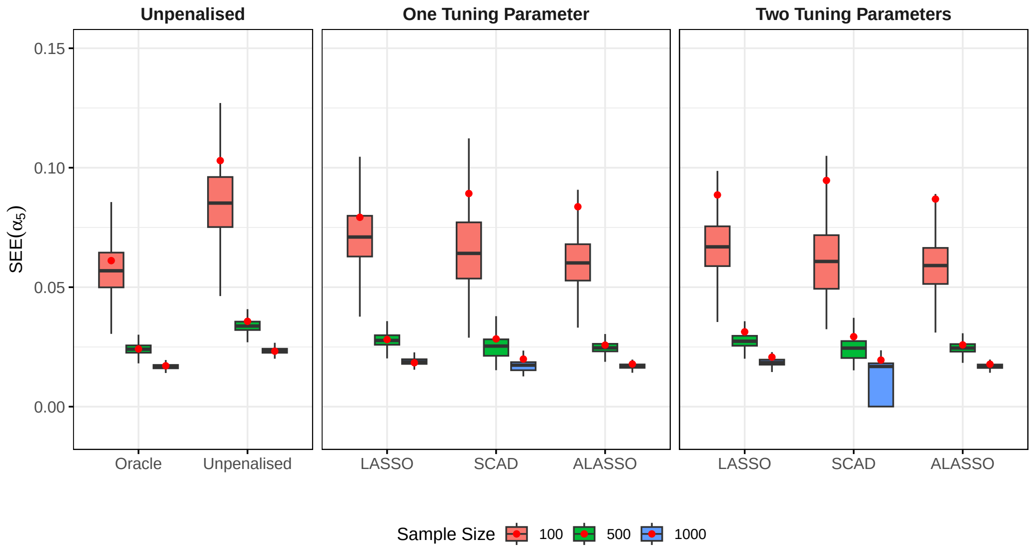


**(b)**

**Figure 6.** (a) $\alpha_5$ coefficient estimates and (b) the corresponding estimated standard error (SEE) and standard deviation (SE) by model and sample size across 1000 replicates (the dashed line across the plots in panel (a) represents the true coefficient value and the dot in panel (b) is for the SE).

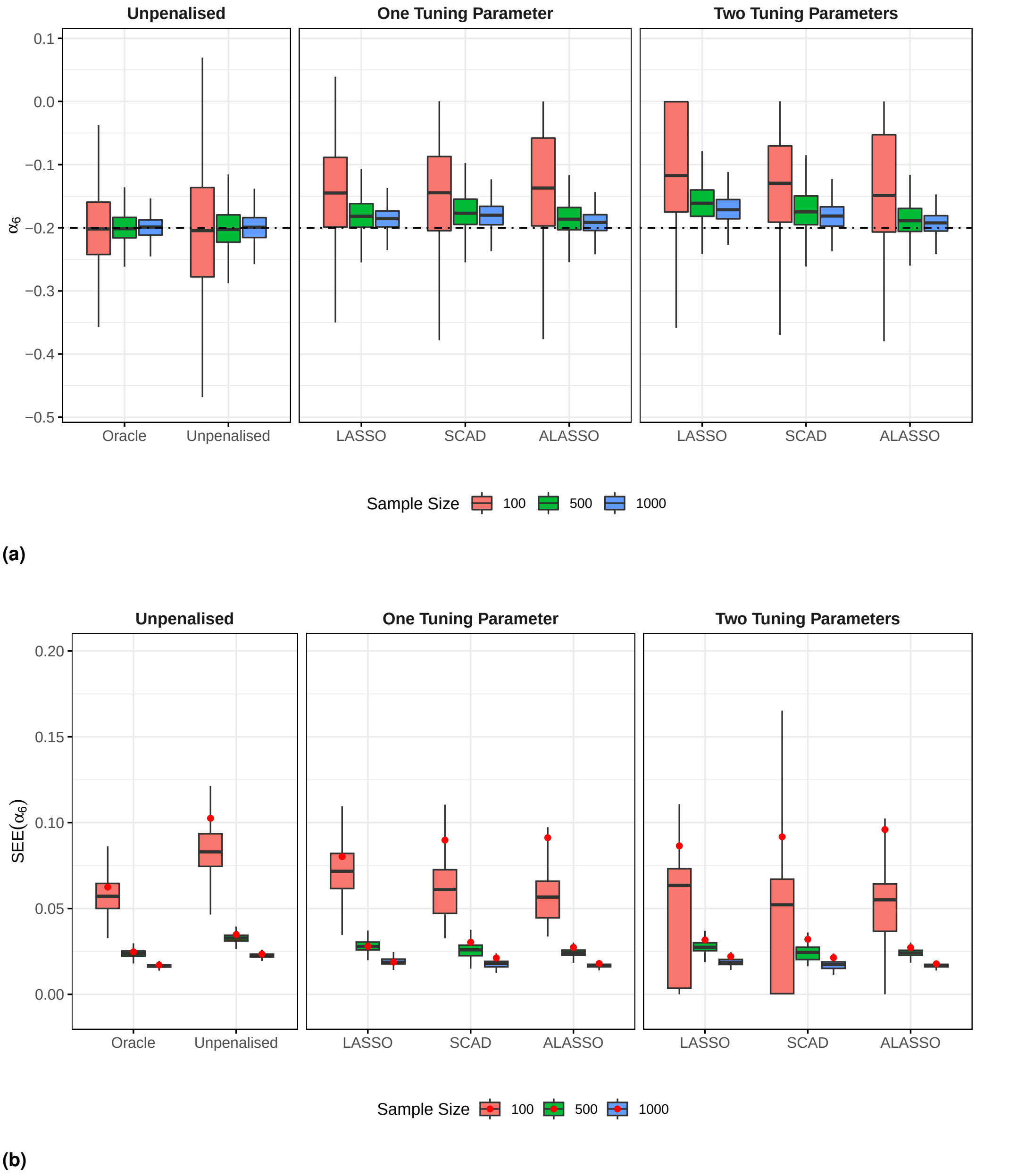


**Figure 7.** (a) $\alpha_6$ coefficient estimates and (b) the corresponding estimated standard error (SEE) and standard deviation (SE) by model and sample size across 1000 replicates (the dashed line across the plots in panel (a) represents the true coefficient value and the dot in panel (b) is for the SE).

## 3 Simulations with 50% censoring

This section displays results from the same simulation scenario described in the main paper but with the censoring increased to 50%.

**Table 4.** Selection results for 50% censoring: variable selection metrics averaged over 1000 simulation replicates.

| One Tuning Parameter | | | | | | | | | | | | | |
|---|---|---|---|---|---|---|---|---|---|---|---|---|---|
| | | LASSO | | | | SCAD | | | | ALASSO | | | |
| $p_{\text{cen}}$ = 50% | $n$ | C(7) | IC(0) | PT | MSE | C(7) | IC(0) | PT | MSE | C(7) | IC(0) | PT | MSE |
| Scale ($\beta$) | 100 | 5.65 | 0.42 | 0.12 | 0.52 | 6.41 | 0.25 | 0.49 | 0.42 | 6.19 | 0.21 | 0.38 | 0.32 |
| | 500 | 5.74 | 0.00 | 0.29 | 0.11 | 6.95 | 0.00 | 0.96 | 0.03 | 6.82 | 0.00 | 0.84 | 0.03 |
| | 1000 | 5.92 | 0.00 | 0.34 | 0.06 | 6.99 | 0.00 | 0.99 | 0.01 | 6.93 | 0.00 | 0.93 | 0.02 |
| Shape ($\alpha$) | 100 | 4.37 | 0.36 | 0.03 | 0.10 | 5.14 | 0.40 | 0.07 | 0.10 | 6.25 | 0.56 | 0.24 | 0.09 |
| | 500 | 4.31 | 0.00 | 0.05 | 0.01 | 6.29 | 0.00 | 0.49 | 0.01 | 6.83 | 0.00 | 0.84 | 0.01 |
| | 1000 | 4.34 | 0.00 | 0.05 | 0.01 | 6.72 | 0.00 | 0.76 | 0.00 | 6.92 | 0.00 | 0.92 | 0.00 |
| Two Tuning Parameters | | | | | | | | | | | | | |
| | | LASSO | | | | SCAD | | | | ALASSO | | | |
| $p_{\text{cen}}$ = 50% | $n$ | C(7) | IC(0) | PT | MSE | C(7) | IC(0) | PT | MSE | C(7) | IC(0) | PT | MSE |
| Scale ($\beta$) | 100 | 5.25 | 0.31 | 0.10 | 0.46 | 6.20 | 0.22 | 0.49 | 0.46 | 6.32 | 0.25 | 0.40 | 0.33 |
| | 500 | 5.35 | 0.00 | 0.20 | 0.09 | 6.83 | 0.00 | 0.93 | 0.03 | 6.87 | 0.00 | 0.88 | 0.03 |
| | 1000 | 5.55 | 0.00 | 0.24 | 0.05 | 6.85 | 0.00 | 0.95 | 0.01 | 6.95 | 0.00 | 0.95 | 0.01 |
| Shape ($\alpha$) | 100 | 5.32 | 0.58 | 0.05 | 0.11 | 5.34 | 0.44 | 0.09 | 0.11 | 6.32 | 0.58 | 0.24 | 0.09 |
| | 500 | 5.59 | 0.02 | 0.24 | 0.02 | 6.32 | 0.01 | 0.52 | 0.01 | 6.84 | 0.00 | 0.85 | 0.01 |
| | 1000 | 5.77 | 0.00 | 0.31 | 0.01 | 6.71 | 0.00 | 0.79 | 0.00 | 6.93 | 0.00 | 0.93 | 0.00 |

**Table 5.** LASSO inferential results for 50% censoring: estimates, standard errors, and confidence intervals.

| LASSO | | | | | | | | | | | | | |
|---|---|---|---|---|---|---|---|---|---|---|---|---|---|
| One Tuning Parameter | | | | | | | | | | | | | |
| $p_{\text{cen}}$ = 50% | | $n$ = 100 | | | | $n$ = 500 | | | | $n$ = 1000 | | | |
| | $\theta$ | $\hat{\theta}$ | SE | SEE | CP | $\hat{\theta}$ | SE | SEE | CP | $\hat{\theta}$ | SE | SEE | CP |
| $\beta_0$ | −1.50 | −1.28 | 0.28 | 0.21 | 0.69 | −1.35 | 0.10 | 0.09 | 0.62 | −1.38 | 0.07 | 0.07 | 0.54 |
| $\beta_1$ | −1.00 | −0.74 | 0.27 | 0.18 | 0.62 | −0.87 | 0.08 | 0.08 | 0.58 | −0.90 | 0.06 | 0.05 | 0.51 |
| $\beta_7$ | −0.80 | −0.48 | 0.25 | 0.17 | 0.53 | −0.63 | 0.09 | 0.08 | 0.42 | −0.67 | 0.06 | 0.05 | 0.36 |
| $\beta_8$ | 0.50 | 0.21 | 0.22 | 0.11 | 0.50 | 0.34 | 0.08 | 0.07 | 0.40 | 0.38 | 0.06 | 0.05 | 0.37 |
| $\alpha_0$ | 0.50 | 0.44 | 0.16 | 0.12 | 0.86 | 0.45 | 0.05 | 0.05 | 0.85 | 0.46 | 0.04 | 0.04 | 0.82 |
| $\alpha_1$ | 0.40 | 0.31 | 0.11 | 0.08 | 0.82 | 0.37 | 0.03 | 0.03 | 0.89 | 0.38 | 0.02 | 0.02 | 0.88 |
| $\alpha_5$ | 0.40 | 0.30 | 0.12 | 0.10 | 0.77 | 0.36 | 0.04 | 0.04 | 0.84 | 0.37 | 0.03 | 0.03 | 0.81 |
| $\alpha_6$ | −0.20 | −0.11 | 0.10 | 0.07 | 0.66 | −0.16 | 0.04 | 0.04 | 0.83 | −0.17 | 0.03 | 0.03 | 0.84 |
| Two Tuning Parameters | | | | | | | | | | | | | |
| $p_{\text{cen}}$ = 50% | | $n$ = 100 | | | | $n$ = 500 | | | | $n$ = 1000 | | | |
| | $\theta$ | $\hat{\theta}$ | SE | SEE | CP | $\hat{\theta}$ | SE | SEE | CP | $\hat{\theta}$ | SE | SEE | CP |
| $\beta_0$ | −1.50 | −1.32 | 0.29 | 0.22 | 0.75 | −1.38 | 0.10 | 0.10 | 0.72 | −1.40 | 0.07 | 0.07 | 0.66 |
| $\beta_1$ | −1.00 | −0.80 | 0.26 | 0.18 | 0.69 | −0.89 | 0.08 | 0.08 | 0.69 | −0.92 | 0.06 | 0.05 | 0.64 |
| $\beta_7$ | −0.80 | −0.57 | 0.27 | 0.17 | 0.65 | −0.68 | 0.09 | 0.08 | 0.59 | −0.70 | 0.06 | 0.05 | 0.54 |
| $\beta_8$ | 0.50 | 0.28 | 0.24 | 0.13 | 0.65 | 0.38 | 0.08 | 0.07 | 0.58 | 0.41 | 0.06 | 0.05 | 0.54 |
| $\alpha_0$ | 0.50 | 0.47 | 0.16 | 0.12 | 0.88 | 0.48 | 0.05 | 0.05 | 0.92 | 0.48 | 0.04 | 0.04 | 0.90 |
| $\alpha_1$ | 0.40 | 0.31 | 0.11 | 0.08 | 0.78 | 0.37 | 0.03 | 0.03 | 0.86 | 0.38 | 0.02 | 0.02 | 0.83 |
| $\alpha_5$ | 0.40 | 0.27 | 0.13 | 0.09 | 0.66 | 0.34 | 0.05 | 0.04 | 0.68 | 0.36 | 0.03 | 0.03 | 0.60 |
| $\alpha_6$ | −0.20 | −0.08 | 0.10 | 0.05 | 0.46 | −0.14 | 0.05 | 0.04 | 0.64 | −0.15 | 0.03 | 0.03 | 0.59 |

SE, standard deviation of estimates over 1000 replications; SEE, average of estimated standard errors over 1000 replications; CP, the empirical coverage probability of a nominal 95% confidence interval.

**Table 6.** SCAD inferential results for 50% censoring: estimates, standard errors, and confidence intervals.

| SCAD | | | | | | | | | | | | | |
|---|---|---|---|---|---|---|---|---|---|---|---|---|---|
| One Tuning Parameter | | | | | | | | | | | | | |
| $p_{\text{cen}}$ = 50% | | $n$ = 100 | | | | $n$ = 500 | | | | $n$ = 1000 | | | |
| | $\theta$ | $\hat{\theta}$ | SE | SEE | CP | $\hat{\theta}$ | SE | SEE | CP | $\hat{\theta}$ | SE | SEE | CP |
| $\beta_0$ | −1.50 | −1.61 | 0.31 | 0.25 | 0.90 | −1.52 | 0.10 | 0.10 | 0.94 | −1.51 | 0.07 | 0.07 | 0.97 |
| $\beta_1$ | −1.00 | −1.09 | 0.26 | 0.19 | 0.88 | −1.02 | 0.07 | 0.07 | 0.95 | −1.01 | 0.05 | 0.05 | 0.95 |
| $\beta_7$ | −0.80 | −0.85 | 0.31 | 0.18 | 0.81 | −0.82 | 0.08 | 0.07 | 0.93 | −0.81 | 0.05 | 0.05 | 0.95 |
| $\beta_8$ | 0.50 | 0.48 | 0.31 | 0.13 | 0.60 | 0.51 | 0.07 | 0.07 | 0.91 | 0.50 | 0.05 | 0.05 | 0.94 |
| $\alpha_0$ | 0.50 | 0.59 | 0.15 | 0.12 | 0.83 | 0.52 | 0.05 | 0.05 | 0.92 | 0.51 | 0.04 | 0.04 | 0.93 |
| $\alpha_1$ | 0.40 | 0.38 | 0.12 | 0.08 | 0.78 | 0.40 | 0.03 | 0.02 | 0.85 | 0.40 | 0.02 | 0.02 | 0.92 |
| $\alpha_5$ | 0.40 | 0.34 | 0.15 | 0.09 | 0.64 | 0.38 | 0.04 | 0.03 | 0.70 | 0.39 | 0.03 | 0.02 | 0.78 |
| $\alpha_6$ | −0.20 | −0.12 | 0.14 | 0.06 | 0.45 | −0.16 | 0.05 | 0.04 | 0.71 | −0.18 | 0.04 | 0.03 | 0.71 |
| Two Tuning Parameters | | | | | | | | | | | | | |
| $p_{\text{cen}}$ = 50% | | $n$ = 100 | | | | $n$ = 500 | | | | $n$ = 1000 | | | |
| | $\theta$ | $\hat{\theta}$ | SE | SEE | CP | $\hat{\theta}$ | SE | SEE | CP | $\hat{\theta}$ | SE | SEE | CP |
| $\beta_0$ | −1.50 | −1.63 | 0.33 | 0.25 | 0.88 | −1.52 | 0.10 | 0.10 | 0.93 | −1.50 | 0.06 | 0.07 | 0.97 |
| $\beta_1$ | −1.00 | −1.11 | 0.26 | 0.20 | 0.87 | −1.02 | 0.07 | 0.07 | 0.95 | −1.01 | 0.05 | 0.05 | 0.95 |
| $\beta_7$ | −0.80 | −0.88 | 0.30 | 0.18 | 0.79 | −0.81 | 0.08 | 0.07 | 0.90 | −0.80 | 0.05 | 0.05 | 0.92 |
| $\beta_8$ | 0.50 | 0.52 | 0.31 | 0.13 | 0.49 | 0.50 | 0.07 | 0.03 | 0.35 | 0.50 | 0.05 | 0.01 | 0.25 |
| $\alpha_0$ | 0.50 | 0.60 | 0.15 | 0.12 | 0.81 | 0.52 | 0.05 | 0.05 | 0.90 | 0.51 | 0.03 | 0.03 | 0.94 |
| $\alpha_1$ | 0.40 | 0.38 | 0.12 | 0.07 | 0.74 | 0.40 | 0.03 | 0.02 | 0.86 | 0.40 | 0.02 | 0.02 | 0.90 |
| $\alpha_5$ | 0.40 | 0.33 | 0.15 | 0.08 | 0.60 | 0.38 | 0.04 | 0.03 | 0.63 | 0.38 | 0.03 | 0.02 | 0.74 |
| $\alpha_6$ | −0.20 | −0.11 | 0.14 | 0.05 | 0.39 | −0.16 | 0.05 | 0.04 | 0.70 | −0.18 | 0.04 | 0.03 | 0.69 |

SE, standard deviation of estimates over 1000 replications; SEE, average of estimated standard errors over 1000 replications; CP, the empirical coverage probability of a nominal 95% confidence interval.

**Table 7.** ALASSO inferential results for 50% censoring: estimates, standard errors, and confidence intervals.

| ALASSO | | | | | | | | | | | | | |
|---|---|---|---|---|---|---|---|---|---|---|---|---|---|
| One Tuning Parameter | | | | | | | | | | | | | |
| $p_{\text{cen}}$ = 50% | | $n$ = 100 | | | | $n$ = 500 | | | | $n$ = 1000 | | | |
| | $\theta$ | $\hat{\theta}$ | SE | SEE | CP | $\hat{\theta}$ | SE | SEE | CP | $\hat{\theta}$ | SE | SEE | CP |
| $\beta_0$ | −1.50 | −1.43 | 0.28 | 0.23 | 0.85 | −1.47 | 0.10 | 0.10 | 0.92 | −1.48 | 0.07 | 0.07 | 0.95 |
| $\beta_1$ | −1.00 | −0.94 | 0.24 | 0.18 | 0.83 | −0.98 | 0.07 | 0.07 | 0.93 | −0.99 | 0.05 | 0.05 | 0.93 |
| $\beta_7$ | −0.80 | −0.69 | 0.26 | 0.17 | 0.77 | −0.77 | 0.08 | 0.07 | 0.90 | −0.78 | 0.05 | 0.05 | 0.92 |
| $\beta_8$ | 0.50 | 0.35 | 0.24 | 0.13 | 0.72 | 0.45 | 0.08 | 0.07 | 0.86 | 0.47 | 0.05 | 0.05 | 0.87 |
| $\alpha_0$ | 0.50 | 0.52 | 0.15 | 0.12 | 0.88 | 0.50 | 0.05 | 0.05 | 0.95 | 0.50 | 0.04 | 0.04 | 0.95 |
| $\alpha_1$ | 0.40 | 0.34 | 0.11 | 0.07 | 0.84 | 0.39 | 0.03 | 0.03 | 0.94 | 0.40 | 0.02 | 0.02 | 0.95 |
| $\alpha_5$ | 0.40 | 0.31 | 0.13 | 0.08 | 0.72 | 0.38 | 0.04 | 0.04 | 0.87 | 0.39 | 0.03 | 0.03 | 0.90 |
| $\alpha_6$ | −0.20 | −0.10 | 0.12 | 0.05 | 0.49 | −0.17 | 0.04 | 0.04 | 0.82 | −0.18 | 0.03 | 0.02 | 0.88 |
| Two Tuning Parameters | | | | | | | | | | | | | |
| $p_{\text{cen}}$ = 50% | | $n$ = 100 | | | | $n$ = 500 | | | | $n$ = 1000 | | | |
| | $\theta$ | $\hat{\theta}$ | SE | SEE | CP | $\hat{\theta}$ | SE | SEE | CP | $\hat{\theta}$ | SE | SEE | CP |
| $\beta_0$ | −1.50 | −1.45 | 0.28 | 0.23 | 0.87 | −1.48 | 0.10 | 0.10 | 0.93 | −1.49 | 0.06 | 0.07 | 0.96 |
| $\beta_1$ | −1.00 | −0.95 | 0.24 | 0.18 | 0.85 | −0.99 | 0.07 | 0.07 | 0.94 | −0.99 | 0.05 | 0.05 | 0.93 |
| $\beta_7$ | −0.80 | −0.70 | 0.27 | 0.17 | 0.78 | −0.77 | 0.08 | 0.07 | 0.90 | −0.78 | 0.05 | 0.05 | 0.93 |
| $\beta_8$ | 0.50 | 0.36 | 0.25 | 0.13 | 0.73 | 0.46 | 0.08 | 0.07 | 0.88 | 0.48 | 0.05 | 0.05 | 0.88 |
| $\alpha_0$ | 0.50 | 0.52 | 0.15 | 0.12 | 0.89 | 0.51 | 0.05 | 0.05 | 0.95 | 0.50 | 0.04 | 0.04 | 0.95 |
| $\alpha_1$ | 0.40 | 0.35 | 0.11 | 0.07 | 0.83 | 0.40 | 0.03 | 0.03 | 0.94 | 0.40 | 0.02 | 0.02 | 0.94 |
| $\alpha_5$ | 0.40 | 0.32 | 0.13 | 0.08 | 0.72 | 0.38 | 0.04 | 0.04 | 0.90 | 0.39 | 0.03 | 0.03 | 0.91 |
| $\alpha_6$ | −0.20 | −0.11 | 0.12 | 0.05 | 0.48 | −0.18 | 0.04 | 0.04 | 0.86 | −0.19 | 0.03 | 0.02 | 0.89 |

SE, standard deviation of estimates over 1000 replications; SEE, average of estimated standard errors over 1000 replications; CP, the empirical coverage probability of a nominal 95% confidence interval.

## 4 Further ALASSO simulations

### 4.1 Correlation of $\rho = 0.8$

Results are displayed for the simulation setting of the main paper but with $\rho$ increased from 0.5 to 0.8.

**Table 8.** ALASSO selection results with $\rho = 0.8$: variable selection metrics averaged over 1000 simulation replicates.

| | | ALASSO | | | | | | | |
|---|---|---|---|---|---|---|---|---|---|
| | | One Tuning Parameter | | | | Two Tuning Parameters | | | |
| $p_{cen}$ = 25% | $n$ | C(7) | IC(0) | PT | MSE | C(7) | IC(0) | PT | MSE |
| Scale ($\beta$) | 100 | 6.12 | 0.14 | 0.39 | 0.24 | 6.28 | 0.18 | 0.44 | 0.24 |
| | 500 | 6.88 | 0.00 | 0.89 | 0.03 | 6.88 | 0.00 | 0.89 | 0.03 |
| | 1000 | 6.95 | 0.00 | 0.95 | 0.01 | 6.93 | 0.00 | 0.94 | 0.01 |
| Shape ($\alpha$) | 100 | 6.22 | 0.17 | 0.39 | 0.03 | 6.34 | 0.20 | 0.44 | 0.03 |
| | 500 | 6.88 | 0.00 | 0.90 | 0.00 | 6.91 | 0.00 | 0.92 | 0.00 |
| | 1000 | 6.94 | 0.00 | 0.95 | 0.00 | 6.96 | 0.00 | 0.96 | 0.00 |

**Table 9.** ALASSO inferential results with $\rho = 0.8$:: estimates, standard errors, and confidence intervals.

| | | ALASSO | | | | | | | | | | | |
|---|---|---|---|---|---|---|---|---|---|---|---|---|---|
| One Tuning Parameter | | | | | | | | | | | | | |
| $p_{cen}$ = 25% | | $n$ = 100 | | | | $n$ = 500 | | | | $n$ = 1000 | | | |
| | $\theta$ | $\hat{\theta}$ | SE | SEE | CP | $\hat{\theta}$ | SE | SEE | CP | $\hat{\theta}$ | SE | SEE | CP |
| $\beta_0$ | −1.50 | −1.50 | 0.23 | 0.21 | 0.92 | −1.48 | 0.09 | 0.09 | 0.93 | −1.49 | 0.07 | 0.06 | 0.94 |
| $\beta_1$ | −1.00 | −1.01 | 0.17 | 0.14 | 0.88 | −0.99 | 0.06 | 0.05 | 0.93 | −0.99 | 0.04 | 0.04 | 0.92 |
| $\beta_7$ | −0.80 | −0.71 | 0.22 | 0.15 | 0.80 | −0.77 | 0.07 | 0.06 | 0.89 | −0.78 | 0.05 | 0.04 | 0.92 |
| $\beta_8$ | 0.50 | 0.37 | 0.21 | 0.12 | 0.75 | 0.46 | 0.06 | 0.06 | 0.87 | 0.48 | 0.04 | 0.04 | 0.88 |
| $\alpha_0$ | 0.50 | 0.53 | 0.11 | 0.09 | 0.89 | 0.50 | 0.04 | 0.04 | 0.94 | 0.50 | 0.03 | 0.03 | 0.94 |
| $\alpha_1$ | 0.40 | 0.39 | 0.05 | 0.03 | 0.89 | 0.40 | 0.01 | 0.01 | 0.95 | 0.40 | 0.01 | 0.01 | 0.95 |
| $\alpha_5$ | 0.40 | 0.35 | 0.08 | 0.05 | 0.76 | 0.39 | 0.02 | 0.02 | 0.92 | 0.39 | 0.02 | 0.01 | 0.92 |
| $\alpha_6$ | −0.20 | −0.13 | 0.08 | 0.04 | 0.70 | −0.19 | 0.02 | 0.02 | 0.90 | −0.19 | 0.02 | 0.01 | 0.91 |
| Two Tuning Parameters | | | | | | | | | | | | | |
| $p_{cen}$ = 25% | | $n$ = 100 | | | | $n$ = 500 | | | | $n$ = 1000 | | | |
| | $\theta$ | $\hat{\theta}$ | SE | SEE | CP | $\hat{\theta}$ | SE | SEE | CP | $\hat{\theta}$ | SE | SEE | CP |
| $\beta_0$ | −1.50 | −1.51 | 0.23 | 0.21 | 0.92 | −1.49 | 0.09 | 0.09 | 0.94 | −1.49 | 0.07 | 0.06 | 0.94 |
| $\beta_1$ | −1.00 | −1.01 | 0.17 | 0.13 | 0.89 | −0.99 | 0.06 | 0.05 | 0.93 | −1.00 | 0.04 | 0.04 | 0.93 |
| $\beta_7$ | −0.80 | −0.72 | 0.23 | 0.15 | 0.79 | −0.77 | 0.07 | 0.06 | 0.90 | −0.78 | 0.05 | 0.04 | 0.93 |
| $\beta_8$ | 0.50 | 0.38 | 0.22 | 0.12 | 0.77 | 0.47 | 0.06 | 0.06 | 0.89 | 0.48 | 0.04 | 0.04 | 0.90 |
| $\alpha_0$ | 0.50 | 0.54 | 0.11 | 0.09 | 0.89 | 0.50 | 0.04 | 0.04 | 0.93 | 0.50 | 0.03 | 0.03 | 0.94 |
| $\alpha_1$ | 0.40 | 0.39 | 0.05 | 0.03 | 0.88 | 0.40 | 0.01 | 0.01 | 0.95 | 0.40 | 0.01 | 0.01 | 0.94 |
| $\alpha_5$ | 0.40 | 0.35 | 0.08 | 0.05 | 0.75 | 0.39 | 0.02 | 0.02 | 0.93 | 0.39 | 0.01 | 0.01 | 0.93 |
| $\alpha_6$ | −0.20 | −0.14 | 0.09 | 0.04 | 0.71 | −0.19 | 0.02 | 0.02 | 0.90 | −0.19 | 0.01 | 0.01 | 0.92 |

SE, standard deviation of estimates over 1000 replications; SEE, average of estimated standard errors over 1000 replications; CP, the empirical coverage probability of a nominal 95% confidence interval.

## 4.2 One non-zero coefficient

Here the number of non-zero coefficients is reduced from three (out of 10) to just one via the following regression specification:

$$\log(\tau_i) = \boldsymbol{x}_i^T(-1.5, -1.0, 0.0, 0.0, 0.0, 0.0, 0.0, 0.0, 0.0, 0.0, 0.0)^T,$$

$$\log(\gamma_i) = \boldsymbol{z}_i^T(0.5, 0.0, 0.0, 0.0, 0.0, 0.4, 0.0, 0.0, 0.0, 0.0, 0.0)^T.$$

**Table 10.** ALASSO selection results with 1 / 10 non-zero coefficients: variable selection metrics averaged over 1000 simulation replicates.

| | | ALASSO | | | | | | | |
|---|---|---|---|---|---|---|---|---|---|
| | | One Tuning Parameter | | | | Two Tuning Parameters | | | |
| $p_{\text{cen}}$ = 25% | $n$ | C(9) | IC(0) | PT | MSE | C(9) | IC(0) | PT | MSE |
| Scale ($\beta$) | 100 | 8.70 | 0.00 | 0.76 | 0.09 | 8.75 | 0.00 | 0.81 | 0.09 |
| | 500 | 8.96 | 0.00 | 0.97 | 0.01 | 8.95 | 0.00 | 0.96 | 0.01 |
| | 1000 | 8.97 | 0.00 | 0.97 | 0.01 | 8.96 | 0.00 | 0.96 | 0.01 |
| Shape ($\alpha$) | 100 | 8.75 | 0.01 | 0.81 | 0.02 | 8.72 | 0.01 | 0.8 | 0.02 |
| | 500 | 8.96 | 0.00 | 0.96 | 0.00 | 8.94 | 0.00 | 0.95 | 0.00 |
| | 1000 | 8.98 | 0.00 | 0.98 | 0.00 | 8.97 | 0.00 | 0.97 | 0.00 |

**Table 11.** ALASSO inferential results with 1 / 10 non-zero coefficients: estimates, standard errors, and confidence intervals.

| | | ALASSO | | | | | | | | | | | |
|---|---|---|---|---|---|---|---|---|---|---|---|---|---|
| | | One Tuning Parameter | | | | | | | | | | | |
| $p_{\text{cen}}$ = 25% | | $n$ = 100 | | | | $n$ = 500 | | | | $n$ = 1000 | | | |
| | $\theta$ | $\hat{\theta}$ | SE | SEE | CP | $\hat{\theta}$ | SE | SEE | CP | $\hat{\theta}$ | SE | SEE | CP |
| $\beta_0$ | −1.50 | −1.50 | 0.20 | 0.19 | 0.94 | −1.49 | 0.08 | 0.08 | 0.95 | −1.50 | 0.06 | 0.06 | 0.96 |
| $\beta_1$ | −1.00 | −0.96 | 0.15 | 0.14 | 0.92 | −0.98 | 0.06 | 0.06 | 0.93 | −0.99 | 0.04 | 0.04 | 0.94 |
| $\alpha_0$ | 0.50 | 0.52 | 0.09 | 0.09 | 0.94 | 0.50 | 0.04 | 0.04 | 0.94 | 0.50 | 0.03 | 0.03 | 0.96 |
| $\alpha_5$ | 0.40 | 0.36 | 0.08 | 0.06 | 0.84 | 0.39 | 0.02 | 0.02 | 0.92 | 0.40 | 0.02 | 0.02 | 0.95 |
| | | Two Tuning Parameters | | | | | | | | | | | |
| $p_{\text{cen}}$ = 25% | | $n$ = 100 | | | | $n$ = 500 | | | | $n$ = 1000 | | | |
| | $\theta$ | $\hat{\theta}$ | SE | SEE | CP | $\hat{\theta}$ | SE | SEE | CP | $\hat{\theta}$ | SE | SEE | CP |
| $\beta_0$ | −1.50 | −1.51 | 0.20 | 0.19 | 0.94 | −1.49 | 0.08 | 0.08 | 0.95 | −1.50 | 0.06 | 0.06 | 0.96 |
| $\beta_1$ | −1.00 | −0.97 | 0.16 | 0.14 | 0.92 | −0.99 | 0.06 | 0.06 | 0.94 | −0.99 | 0.04 | 0.04 | 0.94 |
| $\alpha_0$ | 0.50 | 0.52 | 0.10 | 0.09 | 0.94 | 0.50 | 0.04 | 0.04 | 0.94 | 0.50 | 0.03 | 0.03 | 0.96 |
| $\alpha_5$ | 0.40 | 0.37 | 0.08 | 0.05 | 0.87 | 0.39 | 0.02 | 0.02 | 0.93 | 0.40 | 0.02 | 0.02 | 0.95 |

SE, standard deviation of estimates over 1000 replications; SEE, average of estimated standard errors over 1000 replications; CP, the empirical coverage probability of a nominal 95% confidence interval.

## 4.3 20 covariates and 6 non-zero coefficients

The number of covariates was increased from 10 to 20, while maintaining the same proportion of non-zero coefficients as in the main paper (30%) by setting six non-zero coefficients:

$$\log(\tau_i) = \boldsymbol{x}_i^T(-1.5, -1.0, 0.4, 0.0, 0.0, 0.0, -0.8, 0.0, 0.0, 0.0, 0.0,\\ 0.0, 0.0, 0.6, 0.0, 0.0, 0.70, 0.0, -0.5, 0.0, 0.0)^T,$$

$$\log(\gamma_i) = \boldsymbol{z}_i^T(0.5,0.4, 0.0, 0.0, 0.0, 0.6, 0.0, 0.0, 0.0, 0.3, 0.0,\\ 0.0, 0.0, -0.2, 0.0, 0.0, 0.0, -0.5, -0.4, 0.0, 0.0)^T.$$

**Table 12.** ALASSO selection results with 20 covariates and 6 non-zero coefficients: variable selection metrics averaged over 1000 simulation replicates.

| | | ALASSO | | | | | | | |
|---|---|---|---|---|---|---|---|---|---|
| | | One Tuning Parameter | | | | Two Tuning Parameters | | | |
| $p_{\text{cen}}$ = 25% | $n$ | C(14) | IC(0) | PT | MSE | C(14) | IC(0) | PT | MSE |
| | 100 | 11.62 | 0.39 | 0.09 | 0.57 | 11.91 | 0.46 | 0.11 | 0.58 |
| Scale ($\beta$) | 500 | 13.72 | 0.00 | 0.77 | 0.05 | 13.63 | 0.00 | 0.72 | 0.05 |
| | 1000 | 13.87 | 0.00 | 0.89 | 0.02 | 13.84 | 0.00 | 0.86 | 0.02 |
| | 100 | 12.13 | 0.30 | 0.18 | 0.10 | 12.26 | 0.29 | 0.20 | 0.10 |
| Shape ($\alpha$) | 500 | 13.74 | 0.00 | 0.79 | 0.01 | 13.84 | 0.00 | 0.87 | 0.01 |
| | 1000 | 13.86 | 0.00 | 0.89 | 0.00 | 13.91 | 0.00 | 0.93 | 0.00 |

**Table 13.** ALASSO inferential results with 20 covariates and 6 non-zero coefficients: estimates, standard errors, and confidence intervals.

| ALASSO | | | | | | | | | | | | | |
|---|---|---|---|---|---|---|---|---|---|---|---|---|---|
| **One Tuning Parameter** | | | | | | | | | | | | | |
| $p_{\text{cen}}$ = 25% | | $n$ = 100 | | | | $n$ = 500 | | | | $n$ = 1000 | | | |
| | $\theta$ | $\hat{\theta}$ | SE | SEE | CP | $\hat{\theta}$ | SE | SEE | CP | $\hat{\theta}$ | SE | SEE | CP |
| $\beta_0$ | −1.50 | −1.44 | 0.31 | 0.22 | 0.82 | −1.44 | 0.09 | 0.09 | 0.87 | −1.46 | 0.07 | 0.06 | 0.87 |
| $\beta_1$ | −1.00 | −0.93 | 0.28 | 0.18 | 0.78 | −0.95 | 0.08 | 0.07 | 0.87 | −0.97 | 0.05 | 0.05 | 0.88 |
| $\beta_2$ | 0.40 | 0.27 | 0.21 | 0.12 | 0.70 | 0.35 | 0.06 | 0.06 | 0.82 | 0.37 | 0.04 | 0.04 | 0.89 |
| $\beta_6$ | −0.80 | −0.78 | 0.23 | 0.15 | 0.82 | −0.77 | 0.06 | 0.06 | 0.92 | −0.78 | 0.04 | 0.04 | 0.89 |
| $\beta_{13}$ | 0.60 | 0.50 | 0.24 | 0.15 | 0.75 | 0.55 | 0.07 | 0.06 | 0.85 | 0.57 | 0.04 | 0.04 | 0.88 |
| $\beta_{16}$ | 0.70 | 0.65 | 0.24 | 0.15 | 0.78 | 0.67 | 0.06 | 0.06 | 0.89 | 0.68 | 0.04 | 0.04 | 0.91 |
| $\beta_{18}$ | −0.50 | −0.43 | 0.25 | 0.14 | 0.76 | −0.46 | 0.06 | 0.06 | 0.88 | −0.48 | 0.04 | 0.04 | 0.90 |
| $\alpha_0$ | 0.50 | 0.55 | 0.15 | 0.10 | 0.81 | 0.49 | 0.04 | 0.04 | 0.95 | 0.49 | 0.03 | 0.03 | 0.92 |
| $\alpha_1$ | 0.40 | 0.38 | 0.09 | 0.06 | 0.82 | 0.40 | 0.02 | 0.02 | 0.95 | 0.40 | 0.01 | 0.01 | 0.94 |
| $\alpha_5$ | 0.60 | 0.58 | 0.08 | 0.06 | 0.88 | 0.60 | 0.02 | 0.02 | 0.95 | 0.60 | 0.01 | 0.01 | 0.95 |
| $\alpha_9$ | 0.30 | 0.25 | 0.09 | 0.05 | 0.75 | 0.29 | 0.02 | 0.02 | 0.94 | 0.30 | 0.01 | 0.01 | 0.95 |
| $\alpha_{13}$ | −0.20 | −0.13 | 0.10 | 0.05 | 0.66 | −0.19 | 0.02 | 0.02 | 0.87 | −0.19 | 0.02 | 0.01 | 0.92 |
| $\alpha_{17}$ | −0.50 | −0.49 | 0.09 | 0.06 | 0.86 | −0.50 | 0.02 | 0.02 | 0.95 | −0.50 | 0.02 | 0.02 | 0.96 |
| $\alpha_{18}$ | −0.40 | −0.39 | 0.11 | 0.07 | 0.82 | −0.40 | 0.03 | 0.03 | 0.94 | −0.40 | 0.02 | 0.02 | 0.95 |
| **Two Tuning Parameters** | | | | | | | | | | | | | |
| $p_{\text{cen}}$ = 25% | | $n$ = 100 | | | | $n$ = 500 | | | | $n$ = 1000 | | | |
| | $\theta$ | $\hat{\theta}$ | SE | SEE | CP | $\hat{\theta}$ | SE | SEE | CP | $\hat{\theta}$ | SE | SEE | CP |
| $\beta_0$ | −1.50 | −1.45 | 0.31 | 0.22 | 0.82 | −1.45 | 0.09 | 0.09 | 0.90 | −1.47 | 0.07 | 0.06 | 0.90 |
| $\beta_1$ | −1.00 | −0.94 | 0.28 | 0.18 | 0.77 | −0.96 | 0.08 | 0.07 | 0.91 | −0.98 | 0.05 | 0.05 | 0.91 |
| $\beta_2$ | 0.40 | 0.28 | 0.22 | 0.11 | 0.67 | 0.36 | 0.06 | 0.06 | 0.87 | 0.38 | 0.04 | 0.04 | 0.90 |
| $\beta_6$ | −0.80 | −0.78 | 0.23 | 0.15 | 0.82 | −0.78 | 0.06 | 0.06 | 0.93 | −0.79 | 0.04 | 0.04 | 0.91 |
| $\beta_{13}$ | 0.60 | 0.50 | 0.24 | 0.14 | 0.75 | 0.56 | 0.06 | 0.06 | 0.89 | 0.58 | 0.04 | 0.04 | 0.90 |
| $\beta_{16}$ | 0.70 | 0.66 | 0.24 | 0.14 | 0.77 | 0.68 | 0.06 | 0.06 | 0.91 | 0.69 | 0.04 | 0.04 | 0.93 |
| $\beta_{18}$ | −0.50 | −0.43 | 0.26 | 0.14 | 0.75 | −0.47 | 0.06 | 0.06 | 0.90 | −0.49 | 0.04 | 0.04 | 0.91 |
| $\alpha_0$ | 0.50 | 0.56 | 0.15 | 0.10 | 0.79 | 0.49 | 0.04 | 0.04 | 0.95 | 0.49 | 0.03 | 0.03 | 0.94 |
| $\alpha_1$ | 0.40 | 0.38 | 0.09 | 0.06 | 0.83 | 0.40 | 0.02 | 0.02 | 0.95 | 0.40 | 0.01 | 0.01 | 0.94 |
| $\alpha_5$ | 0.60 | 0.59 | 0.08 | 0.06 | 0.87 | 0.60 | 0.02 | 0.02 | 0.95 | 0.60 | 0.01 | 0.01 | 0.94 |
| $\alpha_9$ | 0.30 | 0.25 | 0.09 | 0.05 | 0.78 | 0.29 | 0.02 | 0.02 | 0.94 | 0.30 | 0.01 | 0.01 | 0.94 |
| $\alpha_{13}$ | −0.20 | −0.13 | 0.10 | 0.05 | 0.67 | −0.19 | 0.02 | 0.02 | 0.89 | −0.19 | 0.02 | 0.01 | 0.92 |
| $\alpha_{17}$ | −0.50 | −0.49 | 0.09 | 0.06 | 0.86 | −0.50 | 0.02 | 0.02 | 0.95 | −0.50 | 0.02 | 0.02 | 0.96 |
| $\alpha_{18}$ | −0.40 | −0.39 | 0.11 | 0.07 | 0.82 | −0.40 | 0.03 | 0.03 | 0.94 | −0.40 | 0.02 | 0.02 | 0.94 |

SE, standard deviation of estimates over 1000 replications; SEE, average of estimated standard errors over 1000 replications; CP, the empirical coverage probability of a nominal 95% confidence interval.

## 4.4 20 covariates and 2 non-zero coefficients

The number of covariates was increased to 20 and the proportion of non-zero coefficients was set to 10% via two non-zero coefficients:

$$\log(\tau_i) = \boldsymbol{x}_i^T(-1.5, -1.0, 0.0, 0.0, 0.0, 0.0, 0.0, 0.0, 0.0, 0.0, 0.0,$$
$$0.0, 0.0, 0.0, 0.0, 0.0, 0.5, 0.0, 0.0, 0.0, 0.0)^T,$$
$$\log(\gamma_i) = \boldsymbol{z}_i^T(0.5,0.0, 0.0, 0.0, 0.0, 0.0, 0.0, 0.0, 0.0, 0.4, 0.0,$$
$$0.0, 0.0, -0.3, 0.0, 0.0, 0.0, 0.0, 0.0, 0.0, 0.0)^T.$$

**Table 14.** ALASSO selection results with 20 covariates and 2 non-zero coefficients: variable selection metrics averaged over 1000 simulation replicates.

| | | ALASSO | | | | | | | |
|---|---|---|---|---|---|---|---|---|---|
| | | One Tuning Parameter | | | | Two Tuning Parameters | | | |
| $p_{\text{cen}}$ = 25% | $n$ | C(18) | IC(0) | PT | MSE | C(18) | IC(0) | PT | MSE |
| Scale ($\beta$) | 100 | 16.53 | 0.14 | 0.28 | 0.24 | 17.03 | 0.18 | 0.42 | 0.23 |
| | 500 | 17.87 | 0.00 | 0.88 | 0.02 | 17.86 | 0.00 | 0.88 | 0.02 |
| | 1000 | 17.95 | 0.00 | 0.95 | 0.01 | 17.93 | 0.00 | 0.94 | 0.01 |
| Shape ($\alpha$) | 100 | 16.84 | 0.11 | 0.40 | 0.06 | 16.68 | 0.09 | 0.40 | 0.05 |
| | 500 | 17.89 | 0.00 | 0.91 | 0.00 | 17.90 | 0.00 | 0.92 | 0.00 |
| | 1000 | 17.95 | 0.00 | 0.96 | 0.00 | 17.95 | 0.00 | 0.96 | 0.00 |

**Table 15.** ALASSO inferential results with 20 covariates and 2 non-zero coefficients: estimates, standard errors, and confidence intervals.

| | | ALASSO | | | | | | | | | | | |
|---|---|---|---|---|---|---|---|---|---|---|---|---|---|
| | | One Tuning Parameter | | | | | | | | | | | |
| $p_{\text{cen}}$ = 25% | | $n$ = 100 | | | | $n$ = 500 | | | | $n$ = 1000 | | | |
| | $\theta$ | $\hat{\theta}$ | SE | SEE | CP | $\hat{\theta}$ | SE | SEE | CP | $\hat{\theta}$ | SE | SEE | CP |
| $\beta_0$ | −1.50 | −1.44 | 0.21 | 0.19 | 0.90 | −1.47 | 0.09 | 0.08 | 0.93 | −1.48 | 0.06 | 0.06 | 0.92 |
| $\beta_1$ | −1.00 | −0.92 | 0.22 | 0.14 | 0.84 | −0.98 | 0.06 | 0.06 | 0.91 | −0.99 | 0.04 | 0.04 | 0.92 |
| $\beta_{16}$ | 0.50 | 0.35 | 0.20 | 0.11 | 0.69 | 0.47 | 0.05 | 0.05 | 0.88 | 0.48 | 0.04 | 0.03 | 0.90 |
| $\alpha_0$ | 0.50 | 0.51 | 0.12 | 0.09 | 0.89 | 0.50 | 0.04 | 0.04 | 0.94 | 0.50 | 0.03 | 0.03 | 0.93 |
| $\alpha_9$ | 0.40 | 0.33 | 0.09 | 0.06 | 0.76 | 0.39 | 0.02 | 0.02 | 0.91 | 0.40 | 0.02 | 0.02 | 0.94 |
| $\alpha_{13}$ | −0.30 | −0.21 | 0.10 | 0.05 | 0.65 | −0.29 | 0.02 | 0.02 | 0.90 | −0.29 | 0.02 | 0.02 | 0.91 |
| | | Two Tuning Parameters | | | | | | | | | | | |
| $p_{\text{cen}}$ = 25% | | $n$ = 100 | | | | $n$ = 500 | | | | $n$ = 1000 | | | |
| | $\theta$ | $\hat{\theta}$ | SE | SEE | CP | $\hat{\theta}$ | SE | SEE | CP | $\hat{\theta}$ | SE | SEE | CP |
| $\beta_0$ | −1.50 | −1.44 | 0.20 | 0.19 | 0.92 | −1.48 | 0.09 | 0.08 | 0.94 | −1.49 | 0.06 | 0.06 | 0.93 |
| $\beta_1$ | −1.00 | −0.92 | 0.20 | 0.14 | 0.83 | −0.98 | 0.06 | 0.06 | 0.92 | −0.99 | 0.04 | 0.04 | 0.93 |
| $\beta_{16}$ | 0.50 | 0.34 | 0.20 | 0.10 | 0.70 | 0.47 | 0.05 | 0.05 | 0.89 | 0.48 | 0.04 | 0.03 | 0.91 |
| $\alpha_0$ | 0.50 | 0.51 | 0.11 | 0.09 | 0.90 | 0.50 | 0.04 | 0.04 | 0.94 | 0.50 | 0.03 | 0.03 | 0.93 |
| $\alpha_9$ | 0.40 | 0.35 | 0.09 | 0.06 | 0.80 | 0.39 | 0.02 | 0.02 | 0.92 | 0.40 | 0.02 | 0.02 | 0.94 |
| $\alpha_{13}$ | −0.30 | −0.23 | 0.09 | 0.05 | 0.73 | −0.29 | 0.02 | 0.02 | 0.90 | −0.29 | 0.02 | 0.02 | 0.92 |

SE, standard deviation of estimates over 1000 replications; SEE, average of estimated standard errors over 1000 replications; CP, the empirical coverage probability of a nominal 95% confidence interval.

## 5 Other approaches for tuning parameter selection

### 5.1 Model-based (Bayesian) Optimization (MBO)

In this section, we compare `DEOptim` to `mlrMBO` (an implementation of model-based Bayesian optimisation) for the selection of tuning parameters. We first ran `DEoptim` 10 times to select the tuning parameter(s) for the ALASSO on the lung cancer data (analysed in Section 5 of the main paper). Within `mlrMBO`, one must set the maximum run time (i.e., there is a fixed time budget), which we set equal to the average time from the 10 `DEOptim` replicates. Moreover, we applied `DEOptim` with an increasing number of iterations in the set $\{9, 25, 100, 400\}$. We can see from Table 16 that, for the same time-budget, `DEOptim` outperforms `mlrMBO` in terms of achieving lower BIC values on average with much less variability. For example, with a budget of just over 3 minutes (186.7 seconds), `DEOptim` achieves an average BIC of 3822.4 for the case with one tuning parameter, where the replicates only vary with respect to the first decimal place. In contrast, `mlrMBO` achieves an average BIC of 3828.4, where the replicates vary by over 25 units. It is noteworthy that the minimum BIC value from the 10 `mlrMBO` replicates does reach the 3822 value, but `DEOptim` achieves this much more consistently.

### 5.2 Grid search

Here, we consider the use of grid search for tuning parameter selection (as is very common in the literature). The results are displayed in Table 17. We can see that, in the case of one tuning parameter, grid search reaches a BIC value of 3826.2 in over 100 seconds (using 100 grid points), whereas, from Table 16, `DEoptim` achieves BIC values in the range 3822.7 – 3823.5 in under 90 seconds. The situation is even worse for grid search in the case of two tuning parameters where even after nearly 700 seconds (with 400 grid points) a BIC of 3842.1 is achieved, whereas `DEoptim` reaches 3801.7 in less than half of this time. Of course, grid search is a very simplistic procedure that can easily miss the minimum if the grid is not sufficiently fine. Moreover, it also suffers from the curse of dimensionality, which is why the performance drops further in the case of two tuning parameters.

## 6 Further Lung cancer study results

In the main paper, we displayed the estimated coefficients for the ALASSO. In Tables 18 and 19 below, we display the coefficients for all approaches.

**Table 16.** A comparison of `DEoptim` and `mlrMBO` for selecting the tuning parameter(s) for the ALASSO penalty (averaged over 10 runs on the lung cancer data).

| One Tuning Parameters | | | | |
|---|---|---|---|---|
| | `DEoptim` | | `mlrMBO` | |
| Max. Iterations | Time (seconds) | BIC (Min., Max.) | Time (seconds) | BIC (Min., Max.) |
| 9 | 87.9 | 3823.1 (3822.7, 3823.5) | 87.9 | 3828.8 (3822.5, 3851.3) |
| 25 | 186.7 | 3822.4 (3822.2, 3822.6) | 186.7 | 3828.4 (3822.2, 3850.9) |
| 100 | 323.7 | 3822.3 (3822.1, 3822.5) | 323.7 | 3828.3 (3822.2, 3850.7) |
| 400 | 493.0 | 3822.3 (3822.0, 3822.9) | 493.0 | 3834.0 (3822.3, 3852.8) |
| **Two Tuning Parameters** | | | | |
| | `DEoptim` | | `mlrMBO` | |
| Max. Iterations | Time (seconds) | BIC (Min., Max.) | Time (seconds) | BIC (Min., Max.) |
| 9 | 158.0 | 3803.4 (3802.4, 3805.0) | 158.0 | 3815.1 (3801.7, 3860.8) |
| 25 | 297.8 | 3801.7 (3801.6, 3801.8) | 297.8 | 3815.8 (3801.7, 3841.1) |
| 100 | 568.0 | 3801.6 (3801.6, 3801.6) | 568.0 | 3813.6 (3801.6, 3860.8) |
| 400 | 682.2 | 3801.5 (3801.3, 3801.6) | 682.2 | 3809.5 (3801.7, 3860.8) |

**Table 17.** Grid search for selecting the tuning parameter(s) for the ALASSO penalty (on the lung cancer data)

| One Tuning Parameter | | | | | |
|---|---|---|---|---|---|
| One tuning parameter | | | Two tuning parameters | | |
| Grid Points | Time (seconds) | BIC | Grid Points | Time (seconds) | BIC |
| 9 | 8.8 | 3856.8 | 9 ($3 \times 3$) | 8.2 | 3947.9 |
| 25 | 26.6 | 3836.4 | 25 ($5 \times 5$) | 26.4 | 3947.9 |
| 100 | 108.3 | 3826.2 | 100 ($10 \times 10$) | 101.5 | 3856.7 |
| 400 | 435.6 | 3822.6 | 400 ($20 \times 20$) | 679.0 | 3842.1 |

**Table 18.** Coefficients estimates and standard errors for the one tuning parameter setup (lung cancer dataset)

| Covariate | | Scale | | | | Shape | | | |
|---|---|---|---|---|---|---|---|---|---|
| | | No Penalty | LASSO | SCAD | ALASSO | No Penalty | LASSO | SCAD | ALASSO |
| Intercept | | **−3.38 (0.66)** | **−2.66 (0.25)** | **−3.35 (0.21)** | **−3.12 (0.17)** | −0.16 (0.22) | −0.13 (0.09) | −0.05 (0.08) | 0.04 (0.03) |
| Treatment | surgery | **−1.69 (0.83)** | −0.82 (0.50) | **−1.21 (0.26)** | **−0.89 (0.21)** | 0.11 (0.21) | −0.14 (0.19) | 0.00 (0.00) | 0.00 (0.00) |
| | chemotherapy | −0.33 (0.37) | 0.00 (0.00) | 0.00 (0.00) | 0.00 (0.00) | −0.03 (0.15) | −0.14 (0.09) | −0.09 (0.08) | 0.00 (0.00) |
| | radiotherapy | **−0.85 (0.21)** | **−0.44 (0.19)** | **−0.84 (0.26)** | −0.16 (0.10) | **0.22 (0.08)** | 0.09 (0.08) | **0.22 (0.10)** | 0.00 (0.00) |
| | chemo. & radio. | **−3.83 (0.98)** | −0.85 (0.59) | **−3.92 (0.93)** | **−2.30 (0.89)** | **0.77 (0.20)** | 0.06 (0.20) | **0.82 (0.17)** | **0.51 (0.21)** |
| Age group | 50− | **−0.90 (0.43)** | 0.00 (0.00) | 0.00 (0.00) | 0.00 (0.00) | **0.39 (0.16)** | 0.05 (0.06) | 0.03 (0.05) | 0.00 (0.00) |
| | 60− | **−0.94 (0.39)** | 0.00 (0.00) | 0.00 (0.00) | 0.00 (0.00) | **0.40 (0.15)** | 0.05 (0.04) | 0.03 (0.03) | 0.00 (0.00) |
| | 70− | −0.77 (0.39) | 0.00 (0.00) | 0.00 (0.00) | 0.02 (0.08) | **0.31 (0.15)** | 0.00 (0.00) | 0.00 (0.00) | 0.00 (0.00) |
| | $>80$ | −0.78 (0.42) | 0.00 (0.00) | 0.00 (0.00) | 0.00 (0.00) | 0.31 (0.17) | 0.00 (0.00) | 0.00 (0.00) | 0.00 (0.00) |
| WHO status | light work | −0.02 (0.45) | −0.42 (0.27) | 0.00 (0.00) | 0.00 (0.00) | 0.02 (0.12) | 0.13 (0.08) | **0.09 (0.00)** | 0.00 (0.00) |
| | unable to work | 0.84 (0.43) | 0.23 (0.23) | **0.71 (0.10)** | **0.41 (0.10)** | −0.10 (0.13) | 0.03 (0.07) | 0.00 (0.00) | 0.00 (0.00) |
| | $>50\%$ walking | **1.31 (0.44)** | **0.79 (0.18)** | **1.24 (0.17)** | **0.99 (0.11)** | −0.13 (0.14) | 0.00 (0.00) | −0.02 (0.07) | 0.00 (0.00) |
| | bed/chair bound | **1.78 (0.50)** | **1.27 (0.29)** | **1.80 (0.24)** | **1.28 (0.28)** | −0.03 (0.20) | 0.00 (0.00) | 0.00 (0.00) | 0.00 (0.00) |
| Sex | male | 0.03 (0.14) | 0.00 (0.00) | 0.00 (0.00) | 0.00 (0.00) | −0.03 (0.05) | −0.01 (0.04) | 0.00 (0.00) | 0.00 (0.00) |
| Smoking status | current smoker | 0.10 (0.22) | 0.00 (0.00) | 0.00 (0.00) | 0.00 (0.00) | 0.15 (0.08) | 0.09 (0.05) | 0.08 (0.05) | 0.00 (0.00) |
| | ex−smoker | −0.05 (0.23) | 0.00 (0.00) | 0.00 (0.00) | 0.00 (0.00) | 0.17 (0.09) | 0.05 (0.05) | 0.06 (0.05) | 0.00 (0.00) |
| | missing | 0.29 (0.40) | 0.00 (0.00) | 0.00 (0.00) | 0.00 (0.00) | 0.00 (0.00) | 0.00 (0.00) | 0.00 (0.00) | 0.00 (0.00) |
| Cell type | small cell | **0.83 (0.26)** | 0.23 (0.21) | **0.46 (0.15)** | **0.31 (0.12)** | −0.05 (0.10) | 0.11 (0.09) | 0.00 (0.00) | 0.00 (0.00) |
| | adenocarcinoma | 0.28 (0.28) | 0.00 (0.00) | 0.00 (0.00) | 0.00 (0.00) | 0.03 (0.10) | 0.09 (0.05) | 0.06 (0.05) | 0.00 (0.00) |
| | other | 0.32 (0.20) | 0.13 (0.10) | 0.03 (0.10) | 0.00 (0.00) | −0.04 (0.07) | 0.00 (0.00) | 0.00 (0.00) | 0.00 (0.00) |
| Metastases | yes | **1.35 (0.28)** | **0.57 (0.11)** | **0.94 (0.18)** | **0.89 (0.12)** | **−0.19 (0.08)** | 0.00 (0.00) | −0.05 (0.05) | 0.00 (0.00) |
| | unknown | **0.83 (0.30)** | 0.17 (0.19) | **0.43 (0.13)** | **0.53 (0.13)** | −0.14 (0.09) | 0.01 (0.07) | 0.00 (0.00) | 0.00 (0.00) |
| Sodium level | $<136\ mmol/l$ | **0.33 (0.14)** | **0.27 (0.09)** | **0.31 (0.14)** | 0.14 (0.08) | −0.01 (0.05) | 0.00 (0.00) | 0.00 (0.00) | 0.00 (0.00) |
| | missing | −0.77 (0.45) | 0.00 (0.00) | 0.00 (0.00) | 0.00 (0.00) | **0.32 (0.16)** | 0.02 (0.09) | 0.04 (0.09) | 0.00 (0.00) |
| Albumen level | $<35\ g/l$ | **0.65 (0.16)** | **0.44 (0.15)** | **0.50 (0.14)** | **0.36 (0.09)** | −0.10 (0.06) | −0.04 (0.06) | −0.07 (0.06) | 0.00 (0.00) |
| | missing | **0.59 (0.28)** | 0.00 (0.00) | 0.00 (0.00) | 0.00 (0.00) | 0.09 (0.15) | **0.15 (0.07)** | 0.12 (0.07) | 0.00 (0.00) |

**Table 19.** Coefficients estimates and standard errors for the two tuning parameters setup (lung cancer dataset)

| | | Scale | | | | Shape | | | |
|---|---|---|---|---|---|---|---|---|---|
| Covariate | | No Penalty | LASSO | SCAD | ALASSO | No Penalty | LASSO | SCAD | ALASSO |
| Intercept | | **−3.38 (0.66)** | **−3.07 (0.25)** | **−3.62 (0.30)** | **−3.15 (0.17)** | −0.16 (0.22) | 0.02 (0.06) | 0.05 (0.06) | 0.04 (0.03) |
| Treatment | surgery | **−1.69 (0.83)** | **−1.18 (0.24)** | **−1.21 (0.25)** | **−0.98 (0.22)** | 0.11 (0.21) | 0.00 (0.00) | 0.00 (0.00) | 0.00 (0.00) |
| | chemotherapy | −0.33 (0.37) | 0.31 (0.21) | **−0.50 (0.21)** | 0.00 (0.00) | −0.03 (0.15) | 0.00 (0.00) | 0.00 (0.00) | 0.00 (0.00) |
| | radiotherapy | **−0.85 (0.21)** | **−0.37 (0.18)** | **−0.56 (0.19)** | **−0.21 (0.10)** | **0.22 (0.08)** | 0.05 (0.07) | 0.11 (0.07) | 0.00 (0.00) |
| | chemo. & radio. | **−3.83 (0.98)** | **−0.74 (0.23)** | **−1.93 (0.81)** | **−0.63 (0.22)** | **0.77 (0.20)** | 0.00 (0.00) | 0.33 (0.22) | 0.00 (0.00) |
| Age group | 50− | **−0.90 (0.43)** | 0.00 (0.00) | 0.00 (0.00) | 0.00 (0.00) | **0.39 (0.16)** | 0.02 (0.06) | 0.02 (0.06) | 0.00 (0.00) |
| | 60− | **−0.94 (0.39)** | 0.00 (0.00) | 0.00 (0.00) | 0.00 (0.00) | **0.40 (0.15)** | 0.02 (0.04) | 0.02 (0.04) | 0.00 (0.00) |
| | 70− | −0.77 (0.39) | 0.00 (0.00) | 0.00 (0.00) | 0.00 (0.00) | **0.31 (0.15)** | 0.00 (0.00) | 0.00 (0.00) | 0.00 (0.00) |
| | $> 80$ | −0.78 (0.42) | 0.00 (0.00) | 0.00 (0.00) | 0.00 (0.00) | 0.31 (0.17) | −0.01 (0.05) | −0.01 (0.05) | 0.00 (0.00) |
| WHO status | light work | −0.02 (0.45) | −0.20 (0.26) | 0.00 (0.00) | 0.00 (0.00) | 0.02 (0.12) | 0.08 (0.08) | 0.09 (0.05) | 0.00 (0.00) |
| | unable to work | 0.84 (0.43) | **0.40 (0.17)** | **0.66 (0.15)** | **0.44 (0.10)** | −0.10 (0.13) | 0.00 (0.00) | 0.00 (0.00) | 0.00 (0.00) |
| | $> 50\%$ walking | **1.31 (0.44)** | **0.90 (0.19)** | **1.14 (0.16)** | **0.97 (0.11)** | −0.13 (0.14) | 0.00 (0.00) | 0.00 (0.00) | 0.00 (0.00) |
| | bed/chair bound | **1.78 (0.50)** | **1.50 (0.29)** | **1.79 (0.26)** | **1.54 (0.25)** | −0.03 (0.20) | 0.00 (0.00) | 0.00 (0.00) | 0.00 (0.00) |
| Sex | male | 0.03 (0.14) | 0.00 (0.00) | 0.00 (0.00) | 0.00 (0.00) | −0.03 (0.05) | 0.00 (0.00) | 0.00 (0.00) | 0.00 (0.00) |
| Smoking status | current smoker | 0.10 (0.22) | 0.12 (0.08) | 0.22 (0.17) | 0.00 (0.00) | 0.15 (0.08) | 0.00 (0.00) | 0.00 (0.00) | 0.00 (0.00) |
| | ex−smoker | −0.05 (0.23) | 0.00 (0.00) | 0.06 (0.26) | 0.00 (0.00) | 0.17 (0.09) | 0.00 (0.00) | 0.00 (0.00) | 0.00 (0.00) |
| | missing | 0.29 (0.40) | 0.00 (0.00) | 0.00 (0.00) | 0.00 (0.00) | 0.00 (0.19) | 0.00 (0.00) | 0.00 (0.00) | 0.00 (0.00) |
| Cell type | small cell | **0.83 (0.26)** | **0.52 (0.16)** | **0.72 (0.16)** | **0.43 (0.13)** | −0.05 (0.10) | 0.00 (0.00) | 0.00 (0.00) | 0.00 (0.00) |
| | adenocarcinoma | 0.28 (0.28) | 0.14 (0.14) | **0.30 (0.14)** | 0.00 (0.00) | 0.03 (0.10) | 0.00 (0.00) | 0.00 (0.00) | 0.00 (0.00) |
| | other | 0.32 (0.20) | 0.13 (0.10) | 0.24 (0.16) | 0.09 (0.09) | −0.04 (0.07) | 0.00 (0.00) | −0.01 (0.06) | 0.00 (0.00) |
| Metastases | yes | **1.35 (0.28)** | **0.67 (0.12)** | **0.77 (0.12)** | **0.84 (0.12)** | **−0.19 (0.08)** | 0.00 (0.00) | 0.00 (0.00) | 0.00 (0.00) |
| | unknown | **0.83 (0.30)** | **0.26 (0.13)** | **0.35 (0.13)** | **0.41 (0.13)** | −0.14 (0.09) | 0.00 (0.00) | 0.00 (0.00) | 0.00 (0.00) |
| Sodium level | $< 136\ mmol/l$ | **0.33 (0.14)** | **0.30 (0.08)** | **0.32 (0.08)** | **0.24 (0.08)** | −0.01 (0.05) | 0.00 (0.00) | 0.00 (0.00) | 0.00 (0.00) |
| | missing | −0.77 (0.45) | 0.00 (0.00) | 0.00 (0.00) | 0.00 (0.00) | **0.32 (0.16)** | 0.00 (0.00) | 0.00 (0.00) | 0.00 (0.00) |
| Albumen level | $< 35\ g/l$ | **0.65 (0.16)** | **0.46 (0.15)** | **0.59 (0.15)** | **0.37 (0.09)** | −0.10 (0.06) | −0.04 (0.06) | −0.08 (0.06) | 0.00 (0.00) |
| | missing | **0.59 (0.28)** | **0.36 (0.14)** | **0.43 (0.14)** | **0.27 (0.14)** | −0.06 (0.12) | 0.00 (0.00) | 0.00 (0.00) | 0.00 (0.00) |
